# Inequalities in body mass index, diet and physical activity in the UK: Longitudinal evidence across childhood and adolescence

**DOI:** 10.1016/j.ssmph.2021.100978

**Published:** 2021-11-25

**Authors:** Nicolás Libuy, David Bann, Emla Fitzsimons

**Affiliations:** a Centre for Longitudinal Studies, University College London Institute of Education, London, UK; b Institute for Fiscal Studies, London, UK

**Keywords:** Body mass index, Obesity, Childhood, Adolescence, Socioeconomic inequalities, Physical activity, Diet, United Kingdom, BMI, Body Mass Index, MCS, Millennium Cohort Study, OLS, Ordinary Least Squares, SES, Socioeconomic status, FE, Fixed Effects

## Abstract

We use longitudinal data across a key developmental period, spanning much of childhood and adolescence (age 5 to 17, years 2006–2018) from the UK Millennium Cohort Study, a nationally representative study with an initial sample of just over 19,000. We first examine the extent to which inequalities in overweight, obesity, BMI and body fat over this period are consistent with the evolution of inequalities in health behaviours, including exercise and healthy diet markers (i.e., skipping breakfast) (n = 7,220). We next study the links between SES, health behaviours and adiposity (BMI, body fat), using rich models that account for the influence of a range of unobserved factors that are fixed over time. In this way, we improve on existing estimates measuring the relationship between SES and health behaviours on the one hand and adiposity on the other. The advantage of the individual fixed effects models is that they exploit within-individual changes over time to help mitigate biases due to unobserved fixed characteristics (n = 6,883).

We observe stark income inequalities in BMI and body fat in childhood (age 5), which have further widened by age 17. Inequalities in obesity, physical activity, and skipping breakfast are observed to widen from age 7 onwards. Ordinary Least Square estimates reveal the previously documented SES gradient in adiposity, which is reduced slightly once health behaviours including breakfast consumption and physical activity are accounted for. The main substantive change in estimates comes from the fixed effects specification. Here we observe mixed findings on the SES associations, with a positive association between income and adiposity and a negative association with wealth. The role of health behaviours is attenuated but they remain important, particularly for body fat.

## Highlights

• We document inequalities in body weight and behaviours over childhood and adolescence in the UK.
• We confirm the previously documented negative SES gradient in adiposity.
• Healthy diet markers and high levels of exercise are negatively associated with body weight.
• We find a positive but small association between income and adiposity, and a negative association with wealth.
• In fixed effects models, the role of health behaviours is attenuated, but they remain important, particularly for body fat.

## 1 Introduction

It is currently estimated that, in the UK, 1 in 3 children leave primary school either overweight or obese (NHS Digital, 2019a). This is a dramatic rise on previous UK-based generations, where the estimated probabilities of overweight or obesity in cohorts born after the 1980s are two to three times greater than those born before the 1980s (Johnson, Li, Kuh, & Hardy, 2015). There is extensive evidence that children from more disadvantaged backgrounds are at higher risk of obesity compared to their more advantaged peers (Bann, Johnson, Li, Kuh, & Hardy, 2018), and that there are significant long-term effects on individuals’ physical and psychological health (Ebbeling, Pawlak, & Ludwig, 2002), and on education and labour market outcomes (Cawley, 2004; Currie, 2009; Tefft, 2018). Tackling obesity has been a policy priority in the UK since the early 1990s, with increased focus on the childhood period since around the turn of the millennium (Jebb, Aveyard, & Hawkes, 2013).

Childhood obesity is a complex and multi-faceted issue, influenced by several factors, including individual-level behavioural, biological, and social ones, as well as family- and community-level factors (Campbell, 2016). Socioeconomic differences in family- and community-level factors (e.g., parental education and obesogenic environments) have been extensively studied, with evidence that they influence children's nutrition and related behaviours (Danielli et al., 2021, Kininmonth et al., 2021, Martin et al., 2012, Pinard et al., 2012, Walker et al., 2010). Interacting closely with family- and community-level factors, individual behaviours can drive an energy imbalance which is thought to regulate body weight.^1^ This has been highlighted by the World Health Organization (WHO, 2012), and a large body of literature emphasizing an increase in food consumption as important in driving the rise in obesity (see, inter alia, Bleich et al., 2008, Brunello et al., 2014), with some, albeit less, emphasis on physical activity (Griffith, Lluberas, & Lührmann, 2016; Lakdawalla, Philipson, & Bhattacharya, 2005). While extensive research has been conducted in this area, the literature that focusses on the links between SES, individual behaviours (food consumption and physical activity) and obesity is largely based on adjusted cross-sectional associations which may be confounded by unobserved factors, such as preferences and genetic factors (Mackenbach, 2020; Pingault et al., 2021).

This paper uses the UK Millennium Cohort Study (MCS), a rich longitudinal study following a nationally representative cohort of individuals born at the turn of the millennium, to first investigate whether the evolution of SES inequalities over childhood/adolescence in health behaviours is consistent with the evolution in Body Mass Index (BMI). Whilst inequalities in BMI have been documented before (Bann et al., 2018; Barriuso et al., 2015), there has been far less focus on inequalities in behaviours, particularly in the same sample – despite their clear relevance to BMI inequities (Hirvensalo & Lintunen, 2011; Lounassalo et al., 2019; Winpenny, Penney, Corder, White, & van Sluijs, 2017). Second, this study aims to study the links between SES, health behaviours and BMI, using richer models than previously, which account for the influence of a range of unobserved factors that are fixed over time. In this way, we can better measure the relationship between SES and health behaviours on the one hand, and BMI on the other. To do this, the paper exploits changes within individuals over time and estimates individual fixed effects models (Angrist & Pischke, 2009).

Our paper reveals stark SES inequalities in trajectories of excess weight, physical activity, and breakfast consumption, widening from early childhood through adolescence. This is over a period coinciding with a major increased policy focus on childhood obesity, the first two decades of this century. It underlines strong persistence in excess weight throughout childhood and adolescence, with one-third of a generation estimated to be overweight or obese as they enter their prime adult years. We provide evidence from richly adjusted models that breakfast consumption and physical activity are negatively associated with childhood/adolescent obesity (BMI and body fat). Whilst we do not claim that this is a particularly novel insight, our estimates are based on richer data and regression models than used in the extant literature, are probed extensively and are robust across several specifications, and confirm existing estimates based on cross-sectional studies. In particular, they control for unobserved time-invariant family characteristics, thereby reducing the extent of omitted variables bias (Wooldridge, 2001). Alongside this, they control for a host of time-varying factors, including socioeconomic and area-level characteristics. They are also designed to help mitigate issues of reverse causality by studying lagged rather than concurrent health behaviours (details in section 4). Our paper thereby contributes to the literature by providing evidence on inequalities in ‘inputs’ and ‘outputs’, for a contemporaneous nationally representative sample of over ten thousand individuals, across the whole of the key developmental period spanning childhood and adolescence. It provides evidence for BMI, as well as body fat, providing a more complete characterisation of childhood and adolescent adiposity than much of the existing literature.

The paper proceeds as follows. The next section discusses related literature and background. In Section 3, we describe the data and study sample, followed by the empirical strategy in section 4. We present the results in Section 5, discussion in Section 6, and our conclusions in Section 7.

## 2 Background

A large number of studies have reported socioeconomic inequalities in childhood BMI in high-income countries, with children and adolescents from lower SES households typically having higher BMI and an increased risk of obesity (Bann et al., 2018; Chen, Martin, & Matthews, 2006). These associations tend to hold across multiple indicators of SES including income, social class and parental education, and across both sexes (Barriuso et al., 2015). However, evidence on whether these associations are causal in nature is far more limited (Cesarini, Lindqvist, Östling, & Wallace, 2016; Oddo, Nicholas, Bleich, & Jones-Smith, 2016; Watson and MouhcineReimer, 2019). For instance, Cesarini et al. (2016) study the impact of a substantive wealth increase caused by lottery wins – arguably a random shock – finding suggestive evidence for a modest effect on reduced obesity risk at age 18. However, there is widespread consensus that several family- and community-factors are associated with childhood obesity. Living in obesogenic environments, with reduced neighbourhood walkability, fewer public recreation facilities and restricted access to healthy food alternatives, has been associated with an elevated risk of obesity, increased sedentary behaviour and less healthy food consumption (Maguire et al., 2015, Qian et al., 2017, Walker et al., 2010, Xue et al., 2019, Zeng et al., 2019, PHE, 2017).

Another set of studies considers the evolution of inequalities in weight across childhood (Chen et al., 2006; Jansen, Mensah, Nicholson, & Wake, 2013; Wardle, Brodersen, Cole, Jarvis, & Boniface, 2006). For instance, Jansen et al. (2013), focusing on children aged 4–10 in Australia, find that low SES children have a higher risk of persistent overweight during childhood. Howe, Lawlor, and Propper (2013) document socioeconomic inequalities in adiposity across birth to 15 years using a sample born in the early 1990s and based in the south-west of England, revealing inequalities opening up in childhood and remaining stable through adolescence (Howe et al., 2013). There remains a gap in evidence around the current evolution of inequalities across all of childhood and adolescence for the UK as a whole (Fitzsimons & Pongiglione, 2019).

A related literature studies the behaviours which are typically socioeconomically patterned and may directly influence childhood obesity. Dietary behaviours and physical activity have been the focus of this, given they represent both sides of the calorie-expenditure balance equation (Cawley, 2010; Tefft, 2018) and are key levers in many policies (Jebb et al., 2013). The increase in high-dense and nutrient-poor food is often cited as a leading cause of the rise in sugar intake during the preschool period, with high energy density food being associated with increasing body fat from childhood to adolescence (Emmett & Jones, 2015; Griffith et al., 2016, 2020). Markers of dietary behaviour and intake have been widely used to investigate links with BMI in population studies (Riera-Crichton & Tefft, 2014). Regular meal consumption is widely considered an important part of a healthy diet, with regularity of breakfast consumption most extensively studied (Deshmukh-Taskar et al., 2010; Dubois, Girard, Potvin Kent, Farmer, & Tatone-Tokuda, 2009; Rampersaud, Pereira, Girard, Adams, & Metzl, 2005). For instance, skipping breakfast during childhood has been associated with metabolic diseases and lower quality dietary intake (Monzani et al., 2019), increased risk of obesity (Alsharairi & Somerset, 2016; Kelly, Patalay, Montgomery, & Sacker, 2016) and higher body fat mass (Wijtzes et al., 2016), although one study finds no association with obesity (Küpers, de Pijper, Sauer, Stolk, & Corpeleijn, 2014).

Regarding physical activity during childhood and adolescence, evidence from both cross-sectional studies (Ekelund et al., 2004; Jiménez-Pavón, Kelly, & Reilly, 2010; Ness et al., 2007; Steele, van Sluijs, Cassidy, Griffin, & Ekelund, 2009) and large-scale longitudinal studies (Dhar & Robinson, 2016; Griffiths et al., 2016; Riddoch et al., 2009), suggests that children with high levels of physical activity are less likely to be obese. Similar evidence has been found in systematic reviews of randomized trials (Kelley et al., 2017, 2019); however, results from observational studies show weaker relationships (Wilks, Besson, Lindroos, & Ekelund, 2011). A related literature evaluating the role of public policies that incentivize physical activity, e.g., increased physical education at school, finds mixed results, depending on child age. While some observational studies show that increases in time in physical education could reduce obesity in young children (Cawley, Frisvold, & Meyerhoefer, 2013; Packham & Street, 2019), the evidence is somewhat weaker for adolescents (Cawley, Meyerhoefer, & Newhouse, 2007).

The well-established associations between SES, diet, physical activity and childhood and adolescent obesity are, however, potentially biased due to omitted variables that correlate with both health behaviours and obesity, but that are not observed and therefore not controlled for in regression models. For instance, we know from these studies that children from families that undertake relatively little exercise and have poorer quality diets have a higher likelihood of obesity. But we also know that families with lower socioeconomic status are more likely to reside in areas with less opportunity to exercise outdoors (Gordon-Larsen, Nelson, Page, & Popkin, 2006), making them less physically active (Stalsberg & Pedersen, 2010). Cross-sectional associations between diet/physical activity and childhood/adolescent excess weight may reflect the influence of such unobserved factors; this may then impact the policy relevance of evidence obtained. Another issue in cross-sectional studies is reverse causality, rendering the association between diet/physical activity and obesity biased if, for instance, current health behaviours are influenced by past body weight. In this paper, we do not claim to fully resolve these important issues, but we do aim to mitigate them by exploiting variation within individuals over time, using rich longitudinal data from a nationally representative UK study. We focus on the period across childhood and adolescence, a highly important developmental period and moreover one coinciding with a major increased policy focus on childhood obesity in the UK.

## 3 Data and study sample

We use data from the Millennium Cohort Study (MCS), a nationally representative UK-wide study following the lives of 19,517 children born across England, Scotland, Wales and Northern Ireland in 2000–02. The MCS provides multiple anthropometric measures of the participants over time (taken by trained interviewers), alongside detailed information on their families, daily lives, behaviours and experiences. The study has had seven sweeps to date, at ages 9 months, 3, 5, 7, 11, 14 and 17. Our analysis is derived from the third to the seventh sweeps, covering ages 5–17 years. We focus on a sample of singletons, our target population (which is 99% of the overall sample), to keep the sample as homogeneous as possible.^2^

Analytic samples vary depending on the empirical strategy. Multilevel linear regression models, which evaluate inequalities in physical activity and breakfast consumption, include participants with valid BMI over the analysis period and with valid family income, measured between 9 months and 5 years (n = 7,220). The sample in the Ordinary Least Squares (OLS) and Fixed Effects (FE) models, which study the association between BMI and physical activity, breakfast consumption, and SES, includes participants with valid BMI and complete data in covariates used in the analysis, from ages 5–17 years (n = 6,883).^3^ Of 7,229 participants with valid BMI, 4.7% (n = 346) were excluded due to missing values on the covariates (Tables S13–S15 in Supplementary data).^4^ When we estimate the cross-sectional prevalence of obesity and participants’ behaviours over time, we use all available information for each sweep, allowing us to improve the precision of population estimates. Standard errors reflect the features of the MCS sampling design, and cross-sectional and longitudinal sampling weights are used to mitigate potential bias due to attrition (Fitzsimons et al., 2020; Silverwood, Calderwood, Sakshaug, & Ploubidis, 2020; Solon, Haider, & Wooldridge, 2015).^5^

### 3.1 Outcomes

BMI is the main outcome of interest; however, we also provide results using body fat and BMI z-scores to more comprehensively characterise adiposity across childhood (Nuttall, 2015). Participants’ heights were measured in the home using a Leicester height measure stadiometer. Weight and body fat measurements were taken using Tanita scales (BF–522W). All interviewers were trained and accredited in using this equipment (Fitzsimons et al., 2020). BMI is constructed as weight divided by height squared. We classified participants as overweight or obese by comparing their BMI with the reference population that describes the distribution of BMI within the population by age and sex. We based the classification on the UK90 cut-offs (Cole, Freeman, & Preece, 1998), which are more widely used in the UK. Classifications based on the international IOTF classifications are contained in the Supplementary data. BMI z-scores are calculated using the zanthro Stata program (Vidmar, Cole, & Pan, 2013). Body fat percentage was calculated by measuring the amount of resistance encountered by a weak electrical current as it travels through the body (Chaplin Grey, Gatenby, & Huang, 2010).

### 3.2 Key exposures

The key exposures in our analysis are SES, physical activity and breakfast consumption. SES is characterised using household income and housing tenure. We use equivalised weekly net family income from ages 5 to 14. The equivalised income adjusts total net income by household size according to the OECD equivalised income scale to provide a measure of net disposable income (Johnson et al., 2012). Income variables are deflated to reflect 2001 prices using the Consumer Prices Index (CPI).^6^ Additionally, housing tenure is included because it is regarded as an important indicator of family wealth (Nasim, 2020).

Regarding exercise, at ages 5, 7 and 11, physical activity (e.g. swimming, gymnastics, football, dancing) was reported by parents, whilst from age 14, weekly moderate to vigorous physical activity was reported by participants (Davies, Frank, McBride, & Calderwood, 2019).^7^ We classified participants’ physical activity using three categories: those who reported not exercising at all (never), exercising 1–4 days per week (irregular) and exercising 5 or more days per week (regular).

We use breakfast consumption as a marker of healthy diet behaviours due to its known association with overall diet quality (Deshmukh-Taskar et al., 2010; Rampersaud et al., 2005; Szajewska & Ruszczyński, 2010; Wijtzes et al., 2015), and the fact that it is consistently recorded in the MCS during the period analysed. Whilst we acknowledge that it is somewhat limited, we provide evidence that it is associated with other dietary behaviours which may influence BMI (Tables S5, S6 and Fig. S1 in Supplementary data). The regularity of breakfast consumption is used to capture changes in eating behaviours from childhood to adolescence. Eating breakfast every day of the week is considered to be ‘regular’ consumption, with ‘irregular’ capturing some days but not all, and the category ‘never’ picking up those who skip breakfast.

### 3.3 Covariates

Time-invariant covariates used in our analyses include sex at birth, six ethnic categories (White, Mixed, Indian, Pakistani and Bangladeshi, Black or Black British, Other ethnic group), region and rural status – all measured at age 5. Region includes the nine Government Office regions in England (North East, North West, Yorkshire and the Humber, East Midlands, West Midlands, East of England, London, South East, South West) and binary indicators for Wales, Scotland and Northern Ireland. The definition of rural status varies by country, reflecting official government classifications.^8^ We derive permanent family income during early childhood using the average equivalised weekly net family income at 9 months, 3 and 5 years. Quintiles of permanent income are created for the purpose of the multilevel linear regression analyses to describe inequality across childhood and adolescence.

A range of time-varying covariates are included to further reduce omitted variable bias. The number of siblings living with the participant is a measure of family structure. We control for a combined measure of labour market status within the household, constructed using information on the labour market status of the main respondent and main respondent's partner (where present) (Fitzsimons & Pongiglione, 2019). We derive the following six categories: both in work; main in work, partner not in work; partner in work, main not in work; both not in work; main in work or on leave, no partner present; and main not in work or on leave, no partner present. We also control for participant age in years at interview and sweep binary indicators.

We also include time-varying socioeconomic factors, measured at the area-level, and which may affect both behaviours and BMI. For this, we used external data on annual unemployment rates at the local authority level over time and linked them to the MCS data using participants’ postcodes at the time of the interview.^9^ More deprived areas in the UK, which are characterised among other factors by higher unemployment rates, are known to concentrate more fast food and other unhealthy food outlets (Fraser & Edwards, 2010; Macdonald, Cummins, & Macintyre, 2007; Maguire et al., 2015), to have a less walkable built environment (Kenyon & Pearce, 2019), and fewer greenspaces (PHE, 2020), all of which are highly relevant to the outcomes under investigation.

## 4 Empirical strategy

### 4.1 Descriptives

We first present descriptive evidence of socioeconomic inequalities in obesity, physical activity and markers of a healthy diet during childhood and adolescence, focusing on measures consistently recorded from ages 5 to 17. We split the sample by quintiles of permanent family income (measured from ages 9 months to age 5) and estimate the proportion of participants classified as underweight, normal weight, overweight and obese. Descriptive statistics for BMI and body fat over time are also estimated. Similarly, we estimate the proportions of participants in the different physical activity and eating behaviour categories over time, both overall and by quintiles of permanent family income.

We then examine whether inequalities in obesity and health-related behaviours have remained stable, widened or narrowed from childhood through adolescence. To do this, we estimate multilevel linear regression models, transforming quintiles of permanent family income to ridit scores^10^, which allows us to measure the absolute difference in outcomes between the lowest and highest income quintiles (Bann et al., 2018). To test whether systematic absolute inequalities change across childhood and adolescence, we include an interaction term between age and the ridit score. Outcomes (level 1) were modelled nested within participants (level 2), and we specified a random intercept and a random slope.

### 4.2 Methods

We first estimate OLS models to examine the relationship between SES and weight. We then add health behaviours to the models, both to examine the extent to which SES inequalities are explained by behavioural factors, and to measure directly the association between health behaviours and weight. Of course, the shortcoming of the OLS estimates is that they may be biased due to the presence of unobserved factors correlated with both the regressors and the outcome of interest. In particular, we expect the OLS estimates measuring the association between positive health behaviours and BMI to be downward biased. This is because unobserved factors, such as general preferences for a healthy lifestyle, are positively correlated with good health behaviours and negatively correlated with BMI. So the coefficient on health behaviours will reflect this, and will overestimate the strength of the negative association with BMI. For this reason, we reduce the influence of such unobserved variables by ‘eliminating’ them from the regression through estimating within-person (fixed effects) models.

The fixed effects model specification is shown below. As there is just one child sampled per household (the ‘participant’), this is analogous to household fixed effects.

(1) $$y_{it}=\beta_{0}+\beta_{1}P_{it-1}+\beta_{2}E_{it-1}\;+X_{it-1}^{\prime }\beta +\alpha_{i}+\delta_{t}+\varepsilon_{it}$$

where i denotes the child, t denotes time (t = 1 denotes age 7/sweep 4, and t = 4 denotes age 17/sweep 7). The fixed effect is represented by α_i_, capturing unobserved time-invariant child and household characteristics. Additionally, y_it_ is child BMI (or body fat in separate regressions); and P_it-1_ and E_it-1_ are prior measures of physical activity and breakfast consumption, lagged one period (see below). X_it-1_ is a vector of time-varying characteristics, including the lag of deflated family income, the lag of housing tenure and the lag of labour market status within the household. We also control for family structure (number of siblings), child age in years at the interview, and annual local-authority unemployment rates; δ_t_ is a survey-round dummy; and $\varepsilon_{it}$ is an *iid* error term.

In order to be able to interpret the fixed effects estimates as causal, we rely on four key assumptions. The first is that there are no unobserved time-varying factors simultaneously affecting exercise and children's BMI, or diet and children's BMI. We mitigate this issue by controlling for a rich set of time-varying factors as described in Section 3.3. A second assumption relates to reverse causation; in other words, we assume that behaviours affect BMI and not the other way round. To make this more plausible, we use behaviours measured in the period prior to observing outcomes. The third and fourth assumptions are that past BMI does not directly affect current BMI, and current behaviours do not affect current BMI (Imai & Kim, 2019). So whilst we do not claim to satisfy all these assumptions, we deal with them to the extent possible using the available data. In Fig. S2 of the Supplementary Data we depict these assumptions via a directed acyclic graph (DAG). Table S16 of Supplementary Data shows results that relax the third and fourth assumptions.

## 5 Results

### 5.1 Descriptives

#### 5.1.1 Subsection: Inequalities in weight

Table S1 in Supplementary Data shows the prevalence of underweight, overweight and obesity throughout childhood and adolescence. Using UK90 cut points, we find that the prevalence of overweight at age 17 is 14.1% (95% confidence interval: 12.9, 15.4) and of obesity is 21.6% (19.9, 23.3).^11^ These proportions are very similar to when participants were previously measured at age 14, when the prevalence of overweight was 14.5% (13.7, 15.3), and of obesity 20.6% (19.6, 21.6). Comparing prevalence by sex, we observe high levels of obesity in both sexes, with a sharp increase between ages 7 and 11 for both, remaining high thereafter. By age 17, 21.0% (18.7, 23.2) of females and 22.2% (19.6, 24.7) of males were obese.

Prevalences of underweight, overweight and obesity by quintile of permanent family income are shown in Table S2. An examination of the prevalence of excess weight at age 17 by family income reveals little evidence of differences in overweight prevalence across income quintiles, but substantial differences in obesity prevalence. Those in lower income groups had a progressively increased risk of obesity: 28.7% (22.5, 34.9) and 29.5% (24.1, 34.9) of those in the lowest and second lowest quintiles respectively are estimated to be obese, compared with 13.9% (12.1, 15.7) in the highest income quintile.

#### 5.1.2 Subsection: Inequalities in health behaviours

Tables S3 and S4 show the prevalence of breakfast consumption and physical activity categories by quintiles of permanent family income. Overall estimates are shown in Tables S9 and S10, in Supplementary data. Although we find that as children get older, they tend to reduce their frequency of regularly eating breakfast, inequalities between income groups widen throughout childhood and adolescence. At age 17, 54.7% (51.5, 57.9) of those in the highest income group reported eating breakfast every day of the week, compared with 31.1% (20.3, 41.8) of those in the lowest income quintile. Regarding physical activity, we find differences between children in the lowest and highest income quintiles at ages 7 and 11, reducing at age 14 and no longer present by age 17.

#### 5.1.3 Subsection: Multilevel regression models

**Fig. 1:**
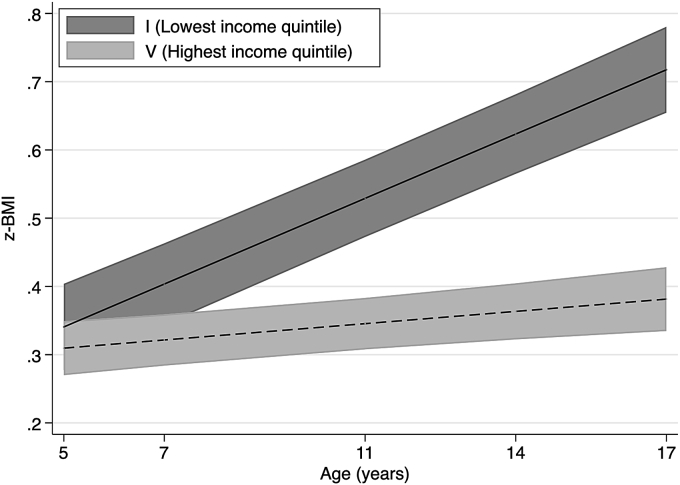
z-BMI across childhood to adolescence by permanent income quintile. Notes: Lines shows the estimated z-BMI and widths of the shaded area are 95% CIs at each age, estimated with multilevel general linear regression models (Table S11 in Supplementary data). z-BMI = body mass index z-scores. Income quintiles characterised by permanent income during childhood based on the average equivalised weekly net family income recorded in the first three sweeps, from 9 months to 5 years.

**Fig. 2:**
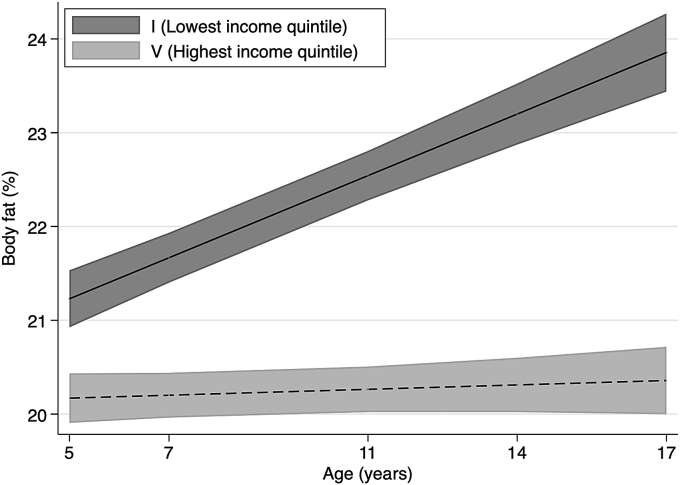
Body fat (%) across childhood to adolescence by permanent income quintile. Notes: Lines shows the estimated body fat percentage and widths of the shaded area are 95% CIs at each age, estimated with multilevel general linear regression models (Table S11 in Supplementary data). Income quintiles characterised by permanent income during childhood based on the average equivalised weekly net family income recorded in the first three sweeps, from 9 months to 5 years.

**Fig. 3:**
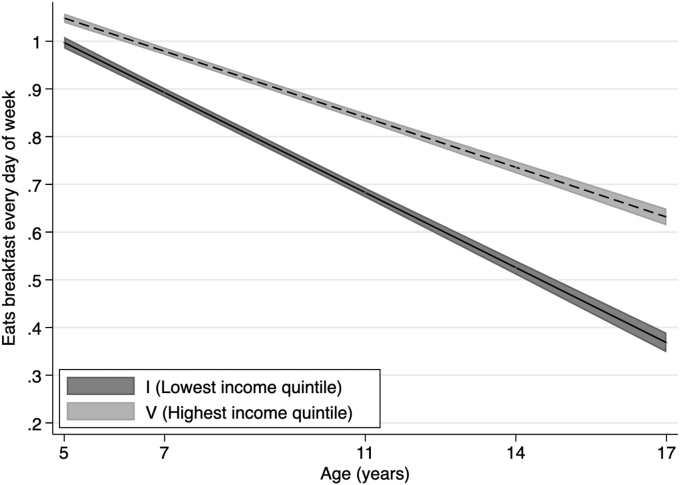
Eats breakfast every day of week across childhood to adolescence by permanent income quintile. Notes: Lines shows the estimated proportion of participants who eats breakfast every day of week and widths of the shaded area are 95% CIs at each age, estimated with multilevel general linear regression models (Table S12 in Supplementary data). Income quintiles characterised by permanent income during childhood based on the average equivalised weekly net family income recorded in the first three sweeps, from 9 months to 5 years.

**Fig. 4:**
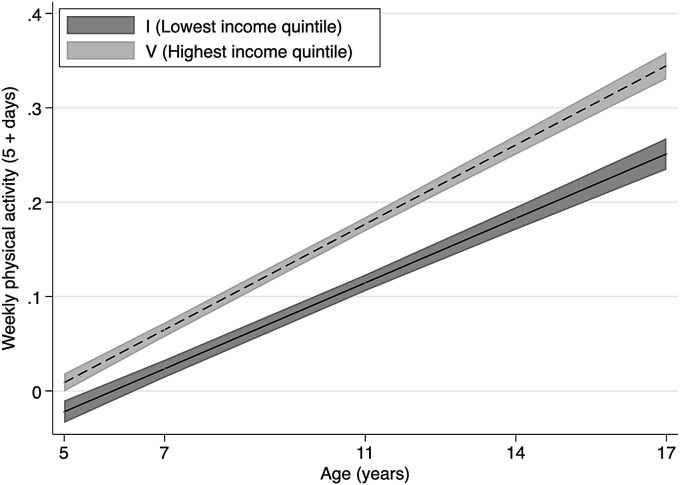
Weekly physical activity (5 + days) across childhood to adolescence by permanent income quintile. Notes: Lines shows the estimated proportion of participants who report 5 or more days of weekly physical activity and widths of the shaded area are 95% CIs at each age, estimated with multilevel general linear regression models (Table S12 in Supplementary data). Income quintiles characterised by permanent income during childhood based on the average equivalised weekly net family income recorded in the first three sweeps, from 9 months to 5 years.

We estimate multilevel regression models in order to evaluate whether inequalities widened or narrowed for childhood and adolescence. We examine income inequalities in trajectories of adiposity, breakfast consumption and physical activity (Fig. 1, Fig. 2, Fig. 3, Fig. 4, details of estimated models are presented in Tables S11–S12 in the Supplementary Data). Fig. 1, Fig. 2, respectively, show the predicted mean of BMI z-scores and body fat percentage from ages 5 to 17 for the lowest and highest income quintile. We find evidence that inequalities by permanent household income widen with age, as indicated by the positive interaction between age and ridit score of permanent family income is in BMI z-scores and body fat models.

Fig. 3 shows the prevalence of breakfast consumption (i.e., eating breakfast every day of the week), estimated using multilevel linear regression models. Fig. 4 shows the corresponding estimated prevalence of regular physical activity (i.e., five or more days of weekly physical activity). Overall, we find that children reduce their consumption of breakfast as they grow older; however, differences in levels between lowest and highest income families show that inequalities in eating behaviours remain stable from childhood until adolescence. We find that inequalities in physical activity widen during childhood and then converge during adolescence (see Table S4).

### 5.2 Main findings

We now turn to multivariable models to study how SES, physical activity and diet are associated with BMI and body fat. Table 1 shows descriptive statistics of variables used in the regression models, measured at age 5, separately by health behaviours (consumption of breakfast, physical activity). Differences in physical activity and breakfast consumption at baseline are observed by socioeconomic status. For instance, among the families of participants who engage in five or more days of physical activity, 86% own a house, compared with 62% among those who never exercise. Similarly, parents of participants who never eat breakfast are more likely to be unemployed than parents of participants who eat breakfast every day.

**Table 1:** Descriptive statistics at age 5 by breakfast consumption and physical activity.

	All		Days per week eats breakfast						Weekly physical activity					
			Never		Some days, but not all days		Every day		Not at all		1–4 days		5 or + days	
Female	50.2	(50.0)	57.7	(50.0)	55.4	(49.8)	49.9	(50.0)	45.6	(49.8)	53.3	(49.9)	44.1	(50.0)
Ethnicity														
White	90.0	(30.0)	86.3	(34.7)	79.2	(40.7)	90.6	(29.2)	84.1	(36.6)	93.7	(24.3)	95.9	(20.0)
Mixed	2.4	(15.4)	4.1	(20.1)	3.8	(19.2)	2.3	(15.1)	2.7	(16.2)	2.3	(15.0)	0.3	(5.2)
Indian	1.9	(13.6)	0.0	(0.0)	2.2	(14.8)	1.9	(13.6)	2.8	(16.4)	1.3	(11.5)	0.9	(9.5)
Pakistani and Bangladeshi	3.1	(17.4)	4.5	(20.9)	9.3	(29.1)	2.8	(16.4)	6.7	(25.1)	0.8	(9.0)	2.5	(15.7)
Black or Black British	1.7	(12.8)	3.9	(19.5)	4.0	(19.5)	1.5	(12.3)	2.4	(15.3)	1.2	(11.0)	0.4	(6.6)
Other Ethnic group	0.9	(9.4)	1.2	(11.1)	1.5	(12.1)	0.9	(9.3)	1.4	(11.7)	0.6	(7.7)	0.0	(0.0)
Urban	76.6	(42.3)	82.3	(38.6)	84.5	(36.2)	76.1	(42.6)	80.9	(39.3)	74.1	(43.8)	63.9	(48.4)
Region														
North East	3.2	(17.5)	0.0	(0.0)	2.7	(16.2)	3.2	(17.6)	3.7	(18.8)	2.8	(16.5)	3.9	(19.5)
North West	9.3	(29.1)	7.3	(26.3)	10.0	(30.0)	9.3	(29.1)	9.4	(29.1)	9.3	(29.0)	12.5	(33.3)
Yorkshire and the Humber	8.7	(28.2)	12.7	(33.6)	10.1	(30.2)	8.6	(28.0)	11.3	(31.6)	7.1	(25.7)	4.2	(20.3)
East Midlands	8.1	(27.3)	11.7	(32.5)	4.5	(20.7)	8.3	(27.6)	8.2	(27.4)	8.0	(27.1)	15.0	(36.0)
West Midlands	7.3	(26.1)	14.6	(35.7)	11.5	(32.0)	7.0	(25.6)	7.7	(26.7)	7.0	(25.5)	11.2	(31.7)
East of England	10.4	(30.5)	16.5	(37.6)	9.5	(29.3)	10.4	(30.5)	10.5	(30.7)	10.3	(30.4)	6.4	(24.6)
London	10.2	(30.2)	7.7	(26.9)	12.4	(33.0)	10.0	(30.1)	10.1	(30.1)	10.2	(30.3)	9.4	(29.3)
South East	16.6	(37.2)	6.6	(25.2)	15.8	(36.5)	16.7	(37.3)	17.0	(37.6)	16.4	(37.0)	12.8	(33.7)
South West	9.9	(29.9)	5.2	(22.5)	8.3	(27.6)	10.0	(30.1)	8.9	(28.5)	10.7	(30.9)	5.1	(22.2)
Wales	5.0	(21.7)	10.5	(31.0)	5.0	(21.8)	4.9	(21.7)	4.2	(20.0)	5.5	(22.8)	2.8	(16.7)
Scotland	8.0	(27.2)	3.9	(19.6)	6.5	(24.6)	8.1	(27.3)	5.2	(22.3)	9.8	(29.7)	11.1	(31.6)
Northern Ireland	3.3	(17.9)	3.3	(18.0)	3.8	(19.2)	3.3	(17.8)	3.8	(19.1)	3.0	(17.0)	5.5	(22.9)
Housing Tenure														
Rent house or other	23.8	(42.6)	48.5	(50.6)	42.3	(49.5)	22.6	(41.8)	38.2	(48.6)	14.7	(35.4)	14.0	(34.9)
Own house	76.2	(42.6)	51.5	(50.6)	57.7	(49.5)	77.4	(41.8)	61.8	(48.6)	85.3	(35.4)	86.0	(34.9)
Combined labour market status														
Both in work	56.3	(49.6)	34.9	(48.2)	43.2	(49.6)	57.1	(49.5)	44.4	(49.7)	64.0	(48.0)	56.3	(49.9)
Only one in work, (main or partner)	27.0	(44.4)	31.2	(46.9)	25.7	(43.8)	27.1	(44.4)	29.9	(45.8)	25.1	(43.4)	30.3	(46.3)
Both not in work	3.3	(17.9)	15.4	(36.5)	6.9	(25.3)	3.0	(17.2)	6.1	(24.0)	1.5	(12.1)	2.7	(16.5)
Main in work or on leave, no partner	7.1	(25.7)	8.3	(27.8)	11.2	(31.6)	6.9	(25.3)	8.2	(27.4)	6.5	(24.6)	5.7	(23.3)
Main not in work nor on leave, no partner	6.3	(24.3)	10.1	(30.5)	13.0	(33.7)	5.9	(23.6)	11.5	(31.9)	3.0	(17.0)	5.0	(22.0)
Number of siblings in household plus participant	1.3	(0.9)	1.5	(1.1)	1.5	(1.1)	1.3	(0.9)	1.5	(1.1)	1.3	(0.8)	1.4	(1.0)
Deflated OECD equivalised weekly family income	388.7	(213.2)	307.8	(219.6)	310.3	(203.3)	393.5	(212.7)	310.5	(183.8)	438.4	(215.7)	441.9	(211.5)
Permanent OECD equivalised weekly family income (0-5y)	380.5	(197.4)	298.4	(229.8)	294.4	(175.4)	385.8	(197.2)	300.7	(171.3)	431.3	(196.5)	432.0	(174.6)
Observations	6883		44		422		6417		2956		3851		76	

Notes: This table show mean/proportions of covariates used in OLS and FE regressions. Standard deviations are shown in parenthesis. Statistics are reported by breakfast consumption and physical activity (see Method section for details).

In Table 2, Table 3, we present our main findings corresponding to OLS and fixed effects estimates from equation (1) for BMI and body fat, respectively. Focusing on BMI, OLS estimates show that the relationship between SES measures (family income, home ownership) and BMI is negative – consistent with previous cross-sectional evidence – and the associations are partly attenuated in OLS models once health behaviours are accounted for (Columns 1 and 2 in Table 2, Table 3). Both SES indicators, family income and owning a house, are negatively associated with BMI. Similar results are found for body fat.

**Table 2:** OLS and FE estimates for BMI.

	(1)		(2)		(3)	
	OLS	95% CI	OLS	95% CI	FE	95% CI
Log of deflated OECD equivalised weekly family income ^a^	−0.27***	[-0.42,-0.11]	−0.23***	[-0.38,-0.08]	0.12**	[0.01,0.23]
Housing Tenure ^a^						
Rent house or other	Ref.		Ref.		Ref.	
Own house	−0.67***	[-0.91,-0.43]	−0.61***	[-0.85,-0.38]	−0.19*	[-0.40,0.01]
Days per week eats breakfast ^a^						
Never (never)			Ref.		Ref.	
Some days, but not all days (irregular)			−0.37	[-0.86,0.11]	−0.02	[-0.33,0.28]
Every day (regular)			−1.03***	[-1.53,-0.53]	−0.15	[-0.46,0.15]
Weekly physical activity ^a^						
Not at all (never)			Ref.		Ref.	
1–4 days (irregular)			−0.04	[-0.19,0.11]	−0.02	[-0.12,0.07]
5 or + days (regular)			−0.37***	[-0.58,-0.16]	−0.13*	[-0.26,0.01]
Combined labour market status ^a^						
Both in work	Ref.		Ref.		Ref.	
Only one in work, (main or partner)	−0.06	[-0.22,0.11]	−0.06	[-0.22,0.11]	0.05	[-0.07,0.16]
Both not in work	−0.11	[-0.58,0.36]	−0.11	[-0.58,0.36]	−0.08	[-0.35,0.20]
Main in work or on leave, no partner	−0.13	[-0.36,0.10]	−0.16	[-0.39,0.07]	0.13	[-0.05,0.31]
Main not in work nor on leave, no partner	−0.07	[-0.39,0.26]	−0.08	[-0.40,0.25]	0.16	[-0.09,0.40]
Number of siblings in household plus participant	−0.14***	[-0.22,-0.05]	−0.14***	[-0.22,-0.05]	−0.09**	[-0.18,-0.01]
Sex						
Female	Ref.		Ref.			
Male	−0.43***	[-0.61,-0.25]	−0.38***	[-0.56,-0.20]		
Sweeps						
Sweep = 7	Ref.		Ref.		Ref.	
Sweep = 11	0.62	[-0.20,1.44]	0.59	[-0.23,1.40]	0.23	[-0.70,1.15]
Sweep = 14	2.09***	[0.65,3.52]	2.00***	[0.57,3.43]	0.9	[-0.55,2.35]
Sweep = 17	3.30***	[1.20,5.40]	3.05***	[0.97,5.14]	1.55	[-0.31,3.41]
Age at interview	0.77***	[0.41,1.14]	0.79***	[0.42,1.16]	0.89***	[0.40,1.38]
Age at interview squared	−0.02**	[-0.03,-0.00]	−0.02**	[-0.03,-0.00]	−0.02*	[-0.03,0.00]
Unemployment rate (LA)	0.08***	[0.04,0.12]	0.07***	[0.03,0.11]	−0.05***	[-0.08,-0.02]
Ethnicity						
White	Ref.		Ref.			
Mixed	−0.1	[-0.65,0.45]	−0.1	[-0.64,0.44]		
Indian	−0.33	[-0.90,0.23]	−0.32	[-0.89,0.25]		
Pakistani and Bangladeshi	−0.19	[-0.53,0.16]	−0.21	[-0.56,0.13]		
Black or Black British	1.57***	[0.83,2.32]	1.52***	[0.77,2.27]		
Other Ethnic group	−0.6	[-1.39,0.18]	−0.58	[-1.36,0.21]		
Rural/Urban						
Urban	Ref.		Ref.			
Rural	−0.04	[-0.24,0.16]	−0.02	[-0.22,0.17]		
Region						
North East	Ref.		Ref.			
North West	−0.73**	[-1.32,-0.15]	−0.74**	[-1.32,-0.16]		
Yorkshire and the Humber	−0.56*	[-1.14,0.03]	−0.56*	[-1.15,0.02]		
East Midlands	−0.50*	[-1.06,0.06]	−0.50*	[-1.06,0.06]		
West Midlands	−0.42	[-1.04,0.19]	−0.43	[-1.04,0.19]		
East of England	−0.66**	[-1.27,-0.04]	−0.66**	[-1.27,-0.06]		
London	−0.74**	[-1.36,-0.13]	−0.73**	[-1.35,-0.12]		
South East	−0.60**	[-1.19,-0.01]	−0.60**	[-1.19,-0.01]		
South West	−0.56*	[-1.16,0.05]	−0.55*	[-1.15,0.05]		
Wales	−0.28	[-0.84,0.28]	−0.3	[-0.85,0.26]		
Scotland	−0.57**	[-1.12,-0.02]	−0.57**	[-1.11,-0.02]		
Northern Ireland	−0.33	[-0.91,0.25]	−0.34	[-0.91,0.24]		
Constant	14.35***	[12.09,16.61]	14.96***	[12.64,17.28]	10.89***	[8.06,13.72]
Individual fixed effect	No		No		Yes	
Observations	27415		27415		27415	

Notes: FE confidence intervals are calculated using cluster standard error at individual level and OLS are calculated using MCS survey design. FE and OLS estimates are calculated using survey weight to account for attrition at age 17. We report 95% confidence intervals in brackets. ***, ** and * denote statistical significance at 1%, 5% and 10% levels respectively. ^a^ Indicates the lag value of the variable (see Method section for details).

**Table 3:** OLS and FE estimates for Body fat (%).

	(1)		(2)		(3)	
	OLS	95% CI	OLS	95% CI	FE	95% CI
Log of deflated OECD equivalised weekly family income ^a^	−0.64***	[-0.97,-0.32]	−0.50***	[-0.81,-0.19]	0.19	[-0.09,0.48]
Housing Tenure ^a^						
Rent house or other	Ref.		Ref.		Ref.	
Own house	−1.31***	[-1.75,-0.88]	−1.14***	[-1.57,-0.71]	−0.48*	[-0.97,0.02]
Days per week eats breakfast ^a^						
Never (never)			Ref.		Ref.	
Some days, but not all days (irregular)			−1.29***	[-2.14,-0.45]	−0.59*	[-1.22,0.05]
Every day (regular)			−2.90***	[-3.74,-2.05]	−1.44***	[-2.08,-0.80]
Weekly physical activity ^a^						
Not at all (never)			Ref.		Ref.	
1–4 days (irregular)			−0.45***	[-0.75,-0.15]	−0.45***	[-0.68,-0.23]
5 or + days (regular)			−1.56***	[-1.97,-1.15]	−0.97***	[-1.32,-0.63]
Combined labour market status ^a^						
Both in work	Ref.		Ref.		Ref.	
Only one in work, (main or partner)	−0.08	[-0.41,0.25]	−0.09	[-0.41,0.24]	−0.02	[-0.30,0.27]
Both not in work	−0.43	[-1.28,0.42]	−0.47	[-1.32,0.38]	−0.29	[-0.97,0.38]
Main in work or on leave, no partner	−0.2	[-0.64,0.24]	−0.29	[-0.72,0.14]	0.18	[-0.27,0.62]
Main not in work nor on leave, no partner	−0.49	[-1.09,0.12]	−0.53*	[-1.13,0.06]	−0.3	[-0.88,0.28]
Number of siblings in household plus participant	−0.32***	[-0.48,-0.16]	−0.33***	[-0.48,-0.17]	−0.16	[-0.36,0.04]
Sex						
Female	Ref.		Ref.			
Male	−7.09***	[-7.42,-6.77]	−6.94***	[-7.26,-6.62]		
Sweeps						
Sweep = 7	Ref.		Ref.		Ref.	
Sweep = 11	0.41	[-1.65,2.48]	0.32	[-1.72,2.37]	2.25*	[-0.06,4.56]
Sweep = 14	2.58	[-0.67,5.84]	2.32	[-0.90,5.53]	4.83***	[1.21,8.45]
Sweep = 17	6.72***	[2.43,11.02]	6.14***	[1.91,10.38]	9.61***	[4.95,14.27]
Age at interview	2.10***	[1.02,3.18]	2.18***	[1.11,3.25]	1.61***	[0.39,2.83]
Age at interview squared	−0.11***	[-0.15,-0.07]	−0.11***	[-0.15,-0.07]	−0.10***	[-0.14,-0.06]
Unemployment rate (LA)	0.18***	[0.09,0.26]	0.16***	[0.08,0.25]	−0.05	[-0.13,0.02]
Ethnicity						
White	Ref.		Ref.			
Mixed	0.31	[-0.72,1.34]	0.31	[-0.70,1.32]		
Indian	1.34**	[0.13,2.56]	1.34**	[0.13,2.55]		
Pakistani and Bangladeshi	1.64***	[0.94,2.34]	1.52***	[0.81,2.23]		
Black or Black British	3.35***	[2.01,4.68]	3.19***	[1.85,4.53]		
Other Ethnic group	−0.13	[-1.64,1.38]	−0.09	[-1.58,1.40]		
Rural/Urban						
Urban	Ref.		Ref.			
Rural	−0.23	[-0.60,0.15]	−0.18	[-0.55,0.20]		
Region						
North East	Ref.		Ref.			
North West	−1.24***	[-2.19,-0.30]	−1.26***	[-2.19,-0.32]		
Yorkshire and the Humber	−1.31***	[-2.22,-0.41]	−1.35***	[-2.24,-0.46]		
East Midlands	−1.01**	[-1.87,-0.15]	−1.03**	[-1.88,-0.18]		
West Midlands	−0.98**	[-1.93,-0.02]	−0.99**	[-1.95,-0.03]		
East of England	−1.45***	[-2.47,-0.44]	−1.49***	[-2.49,-0.50]		
London	−1.68***	[-2.71,-0.66]	−1.66***	[-2.67,-0.64]		
South East	−1.16**	[-2.06,-0.25]	−1.18**	[-2.07,-0.28]		
South West	−1.12**	[-2.03,-0.22]	−1.12**	[-2.02,-0.22]		
Wales	−0.61	[-1.45,0.23]	−0.65	[-1.48,0.18]		
Scotland	−1.24***	[-2.06,-0.42]	−1.23***	[-2.04,-0.42]		
Northern Ireland	−0.57	[-1.47,0.34]	−0.6	[-1.49,0.29]		
Constant	19.82***	[13.66,25.98]	21.44***	[15.19,27.70]	15.68***	[8.61,22.74]
Individual fixed effect	No		No		Yes	
Observations	27415		27415		27415	

Notes: FE confidence intervals are calculated using cluster standard error at individual level and OLS are calculated using MCS survey design. FE and OLS estimates are calculated using survey weight to account for attrition at age 17. We report 95% confidence intervals in brackets. ***, ** and * denote statistical significance at 1%, 5% and 10% levels respectively. ^a^ Indicates the lag value of the variable (see Method section for details).

However, when we control for fixed and unobserved factors, shown in Column 3, the association between household income and BMI becomes positive. The difference is likely explained by the fact that the OLS (between-individual) estimate uses variation across individuals over time and reflects the well-documented negative cross-sectional association between income and BMI. However, this income coefficient also captures unobserved heterogeneity that exists between individuals. The FE estimate instead relies on changes over time *within* individuals. In this way, it relies solely on estimation using within-family change in income and within individual change in BMI; it removes the influence of fixed unobserved confounding factors, and reveals that an increase in family income over time is instead associated with increased BMI. However, it is worth stressing that both the OLS and FE coefficients are relatively small in magnitude and imprecisely estimated, i.e., confidence interval close to null. As such, caution is warranted when interpreting this result. For example, from the FE model, we estimate that a one per cent increase in weekly family income is associated with a 0.0012 point increase in BMI (=0.12 from Table 2 divided by 100). Additionally, the negative association with home ownership, a widely used measure of wealth, remains negative in FE models, both for BMI and body fat. This suggests that increases in household wealth (proxied by home ownership) over time are negatively associated with weight gain throughout childhood and adolescence.

Turning to health behaviours, OLS estimates in Column 2 show a negative association between weight gain (BMI and body fat) and breakfast consumption and exercise. Once the individual fixed factors are accounted for, these associations are attenuated but remain negative. For instance, FE estimates, shown in Column 3, indicate that a higher weekly frequency of breakfast consumption is negatively associated with body fat, but we cannot rule out a null effect for BMI. We estimate that, compared to participants who never eat breakfast, those who eat breakfast regularly have lower percentages of body fat (95% CI: -2.09,-0.81), a 6.7% reduction with respect to the sample mean of 21.6. Over a period of ten years, from 7 to 17, this figure represents an annual average reduction of 0.7% in body fat.^12^ The coefficients for physical activity in FE models indicate a negative association with both BMI and body fat, with higher physical activity during childhood and adolescence reducing adiposity. We observe larger effects in FE models for regular exercise, i.e., five or more days of weekly physical activity, than for irregular weekly exercise.

The fact that the FE specification attenuates the relationship between health behaviours and weight suggests that fixed, unobserved factors that are positively correlated with healthy behaviours – such as familial preferences for a healthy lifestyle – and that also tend to drive down BMI, are relevant factors that may partly confound the relationship between health behaviours and weight, and important to account for in estimating causal parameters of policy relevance.

## 6 Discussion

In a large longitudinal UK representative cohort study covering almost two decades 2000/02–2018/19, we find that SES inequalities in adiposity, breakfast consumption and physical activity exist and widen from childhood to adolescence. From ages 11–17, we estimate that a stable 13% of children in the highest family income quintile are obese, in contrast to an increase from 24% to 29% in the lowest quintile over the same period. As children get older, patterns of healthy diet markers and physical activity change towards less regular breakfast consumption and increased physical activity overall; however, SES inequalities in these well-known drivers of obesity widen during the transition to adolescence. Results from rich multivariable models that seek to address reverse causality and unobserved fixed confounders show that household wealth is negatively associated with weight gains, and household income is positively associated with BMI and body fat, although estimates are relatively small in magnitude. Estimates for breakfast consumption and physical activity remain negatively associated with body mass and body fat after controlling for unobserved fixed confounders, providing robust evidence on the association of two purported behavioural drivers of childhood obesity.

Our finding that the prevalence of overweight and obesity remain high during adolescence and in both sexes is consistent with existing evidence for the UK (NHS Digital, 2019a, NHS Digital, 2019b) and for other European countries (Garrido-Miguel et al., 2019). Further, our results that SES inequalities in overweight/obesity, BMI and body fat widen from childhood to late adolescence (age 17) confirm findings from previous studies focusing on younger children and using different indicators of socioeconomic status than family income (paternal social class and maternal education) (Bann et al., 2018; Barriuso et al., 2015; Howe et al., 2013; Jansen et al., 2013; Rougeaux, Hope, Law, & Pearce, 2017). These trends in obesity are concerning given some evidence that BMI tracks from childhood to adulthood in higher quintiles of the BMI distribution and disproportionately in lower compared to higher socioeconomic families (Norris, Bann, Hardy, & Johnson, 2020).

The evidence that inequalities in weight and health behaviours widen from childhood to adolescence may be explained by inequalities affecting the cumulative nature of obesity risk drivers during this period. There is abundant evidence that inequalities affect the incremental process of academic skill formation (Heckman, 2012), which may resemble the cumulative process of healthy habit formation or other drivers of weight gain during childhood and adolescence (Flodmark, Marcus, & Britton, 2006; Gibson et al., 2012; Issanchou, 2017). The process of healthy habit formation is strongly influenced by structural inequalities around children, such as neighbourhood deprivation, the physical environment, infrastructure of policies and services related to food and physical activity, norms and values, among other factors. The interaction between these upper stream factors and the accumulation of adverse individual-level circumstances over time may explain why differences in healthy behaviours and BMI between more and less deprived children widen as they grow up.

Our FE estimates help reduce omitted variables bias due to time-invariant confounding factors (such as genetic factors (Mackenbach, 2020; Pingault et al., 2021), and time discounting preferences (Barlow, Reeves, McKee, Galea, & Stuckler, 2016)) which simultaneously influence SES, health behaviours, and BMI. Though attenuated, the fact that the estimates remain negatively associated with weight, along with the findings that socioeconomic inequalities in ‘inputs’ mirror inequalities in childhood and adolescent adiposity suggest that interventions to encourage healthy behaviours may reduce the risk of excess weight later in life. The socioeconomic inequalities in physical activity are consistent with international cross-sectional evidence (Bann, Scholes, Fluharty, & Shure, 2019; Brodersen, Steptoe, Boniface, & Wardle, 2007; Elgar et al., 2015; Johnsen, Toftager, Melkevik, Holstein, & Rasmussen, 2017), as well as cross-sectional evidence for early years with the MCS (Love, Adams, Atkin, & van Sluijs, 2019). Similarly, dietary inequalities have been found in the Avon Longitudinal Study of Parents and Children (Emmett & Jones, 2015), which use more detailed dietary measurements. Changes in individual behaviours may play an important role in reducing the risk of obesity and, provided they are effective amongst socioeconomically disadvantaged groups, potentially reduce socioeconomic inequality in obesity (Hillier-Brown et al., 2014).

In contrast to previous studies that have evaluated the role of physical activity (Griffiths et al., 2016; Riddoch et al., 2009) and breakfast consumption (Kelly et al., 2016; Monzani et al., 2019) using between-individual variation, fixed effects models better account for omitted variable bias by exploiting variation over time within individuals. Our finding that increased physical activity is associated with decreased body mass and body fat is consistent with studies based on accelerometers – a more objective measure of physical activity – with several studies finding an inverse association between moderate-to-vigorous physical activity and adiposity during childhood and adolescence (Basterfield et al., 2012; Griffiths et al., 2016; Pate et al., 2013; Riddoch et al., 2009).

Our fixed effects estimates will not measure causal parameters if time-varying residual confounding is present. Whilst we cannot rule out such confounding, we greatly mitigate the extent of this issue using the detailed information in the MCS to control for changes in family socioeconomic conditions and area-level economic conditions that are known to be associated with changes in adiposity and healthy behaviours. There are other time-varying individual factors that were not recorded in the MCS during the period analysed that may bias our results, such as perceptions of body image and mental health. Further research should be conducted to evaluate how these potential confounders may bias the measured associations.

One potential weakness in our study is that although body weight and fat mass were measured at all ages 5, 7, 11, 14 and 17, objective measures of physical activity (e.g., accelerometers) and more detailed dietary composition were not.^13^ For this reason, and based on previous evidence, we proxy changes in healthy behaviours using reported physical activity and breakfast consumption. While we cannot rule out that other unmeasured aspects of time-varying dietary behaviours may be associated with BMI, we provide evidence that breakfast consumption is associated with healthier food consumption (see Tables S5, S6 and Fig. S1 in the Supplementary Data). Another potential bias could arise if measurement error in behaviours varies by socioeconomic status - for example, if individuals from lower income households tend to underreport physical activity, then the association between exercise and BMI would be underestimated. Bias due to non-random attrition is another potential weakness in this study, which we address using inverse probability weights, thereby adjusting for selection on observables (Fitzsimons et al., 2020; Silverwood et al., 2020). Although 4.7% of observations were excluded due to missing values in the covariates used in the analysis, multiple imputation shows that our main results are not substantially changed when they are included (Table S15 in the Supplementary Data).

## 7 Conclusion

Since the turn of the millennium, tackling childhood and adolescent obesity has become a major global policy priority due to the increased risk of excess weight observed in recent decades, affecting disproportionately children from more disadvantaged backgrounds. Further, the evidenced detrimental long-term effects on individuals’ physical, psychological, educational and labour market outcomes makes it imperative to improve our understanding of its causes, thereby contributing to effective policy.

With a representative longitudinal cohort of children born in the early 2000s, our study provides new evidence that inequalities in obesity, physical activity and markers of a healthy diet (i.e., breakfast consumption) open up early on and widen from childhood through adolescence. It provides evidence from rich models that account for an array of observed and unobserved confounders that wealth (home ownership), physical activity and regular breakfast consumption are associated with reduced body weight and body fat over the formative years – a critical but understudied period in terms of drivers of obesity. We show further that SES inequalities in child and adolescent weight remain but are much reduced once health behaviours are accounted for. Our results speak to the current public debate regarding policy-effective measures to tackle the obesity epidemic in younger generations and the extent to which we might expect these policies to reduce socioeconomic inequalities in childhood and adolescent obesity. For instance, fixed effects estimates suggest that housing tenure – an indicator of wealth that is less likely influenced directly by reverse causality – may be causally related to reduced BMI. Finally, our results suggest that promoting medium- or long-term behavioural changes among families from more disadvantaged groups may help to reduce inequalities in obesity and mitigate harmful effects on adult health, social and economic outcomes. The promotion of healthy behaviours, however, should be considered within the context of other structural and social determinants of obesity risks, to the extent that they shape and limit families’ decisions over individual behaviours.

## Ethical statement

The UK Millennium Cohort Study has received ethical approval from the National Health Service (NHS) Research Ethics Committee (REC) system. Ethical approval has been sought for all MCS surveys since the start of the study in 1999.

## Author statement

Bann, Fitzsimons and Libuy were jointly responsible for the conceptualization of the manuscript, methodology, data curation, and formal statistical analysis. Libuy and Fitzsimons contributed equally to the writing of the first draft of the manuscript and led the statistical analysis. The three authors were jointly responsible for reviewing and editing the manuscript.

## Declaration of competing interest

None.

## Footnotes

1 Focusing on only individual-level determinants of childhood obesity has been challenged because it overlooks other community-level factors influencing the risk of obesity. However, it is still considered one major element in the policy agenda. For example, on its recent flagship policy, the UK Govt Childhood Obesity: A Plan for Action (UK Government, 2016), it was noted that “*at its root obesity is caused by an energy imbalance: taking in more energy through food than we use through activity*” (pp 3).

2 We excluded twins and triplets (n = 535 participants) because their health-related maternal characteristics have been found to be systematically different from singletons, which may confound our analyses (Bhalotra & Clarke, 2019).

3 FE models did not include BMI at age 5/sweep 3 in the regression analysis because breakfast consumption and physical activity were not collected at age 3.

4 As a robustness exercise, we use multiple imputation methods to deal with missing observations in covariates used in our main fixed effect models (Supplementary Data, Table S15). Our main FE results do not change substantively.

5 Attrition by the MCS seventh sweep (age 17) is around 45% (Fitzsimons et al., 2020).

6 The factors used to deflate family income were CPI 2001, 2005, 2008, 2012, and 2015. Source: https://www.ons.gov.uk/economy/inflationandpriceindices.

7 According to the Chief Medical Officers' Physical Activity Guidelines, moderate physical activities for Children and Young People (5–18 ages) include walking, cycling and shopping, and vigorous physical activities include playing football, dancing, swimming.

8 Our main results are similar when region and rural status variables are considered time-varying variables. In England, rural was defined using the ONS 2005 Rural Urban Morphology Code, and rural status includes participants living in ‘Town and Fringe’ and in ‘Village, Hamlet & Isolated Dwellings’. In Scotland, rural status is defined using the Scottish Executive Urban Rural Classification. In Northern Ireland, rural status is defined using the Rural Status 2005 Classification and areas classified as ‘Mixed Urban-Rural’ were considered rural.

9 The local-authority (LA) annual unemployment rates of 2008, 2012, 2015 and 2008, were respectively linked to MCS at ages 7, 11, 14 and 17. LA unemployment rates for Great Britain were obtained from NOMIS – Official Labour market Statistics (www.nomisweb.co.uk) and for Northern Ireland were obtained from Northern Ireland Statistics and Research Agency (NISRA – www.nisra.gob.uk). LAs correspond to areas of local government in the United Kingdom, which have varied little over time.

10 Ridit is a method for replacing the categories of an ordinal variable with scores between a predefined range. For example, the average ridit for the lowest income quintile shows the probability that a member of this group differs from a member of the reference population. Ridit scores have been used to provide a single quantification of inequality in multilevel linear regression models (Bann et al., 2018). We use the wridit command in Stata (Newson, 2012).

11 Tables S7 and S8 in supplementary data show the prevalence of obesity over childhood and adolescence by quintiles of permanent family income using the IOTF classification.

12 Assuming the reduction is linear in participant's age, the annual average decline of 0.7% is obtained by dividing 6.7% by ten years.

13 Accelerometery data was collected at ages 7 and 14 only.
