# Supplementary material for: Inequalities in body mass index, diet and physical activity in the UK: Longitudinal evidence across childhood and adolescence
Source: SSM Popul Health. 2021 Nov 25;16:100978. doi: 10.1016/j.ssmph.2021.100978 (PMC8671115; doi:10.1016/j.ssmph.2021.100978)
Supplement: Multimedia component 1 [file mmc1.doc]

**Inequalities in body mass index, diet and physical activity in the UK: longitudinal evidence across childhood and adolescence**

**Supplementary data**

Table S1. Prevalence of obesity over childhood and adolescence

|  |  |  | All |  |  |
| --- | --- | --- | --- | --- | --- |
|  | 5 | 7 | 11 | 14 | 17 |
| Underweight | 0.8 | 1.1 | 1.4 | 1.6 | 1.9 |
|  | [0.6,0.9] | [0.8,1.3] | [1.2,1.7] | [1.3,2.0] | [1.4,2.3] |
| Normal weight | 73.9 | 74.4 | 63.2 | 63.2 | 62.4 |
|  | [73.1,74.7] | [73.4,75.3] | [62.2,64.3] | [62.1,64.3] | [60.6,64.2] |
| Overweight | 14.1 | 11.9 | 15.2 | 14.5 | 14.1 |
|  | [13.5,14.7] | [11.3,12.5] | [14.4,16.0] | [13.7,15.3] | [12.9,15.4] |
| Obese | 11.2 | 12.7 | 20.2 | 20.6 | 21.6 |
|  | [10.6,11.8] | [11.9,13.4] | [19.3,21.1] | [19.6,21.6] | [19.9,23.3] |
|  |  |  | Female |  |  |
|  | 5 | 7 | 11 | 14 | 17 |
| Underweight | 0.6 | 0.9 | 1.8 | 1.1 | 1.3 |
|  | [0.4,0.8] | [0.6,1.2] | [1.4,2.3] | [0.7,1.5] | [0.7,1.9] |
| Normal weight | 76.4 | 75.5 | 64.3 | 62.5 | 62.7 |
|  | [75.3,77.6] | [74.3,76.6] | [62.8,65.8] | [60.8,64.2] | [60.3,65.2] |
| Overweight | 12.8 | 11.9 | 15 | 16 | 15 |
|  | [11.9,13.6] | [11.0,12.8] | [13.9,16.1] | [14.8,17.2] | [13.1,17.0] |
| Obese | 10.2 | 11.8 | 18.9 | 20.3 | 21 |
|  | [9.3,11.0] | [10.8,12.7] | [17.7,20.1] | [18.8,21.9] | [18.7,23.2] |
|  |  |  | Male |  |  |
|  | 5 | 7 | 11 | 14 | 17 |
| Underweight | 0.9 | 1.2 | 1.1 | 2.1 | 2.4 |
|  | [0.7,1.2] | [0.9,1.6] | [0.8,1.3] | [1.6,2.6] | [1.8,3.1] |
| Normal weight | 71.5 | 73.3 | 62.2 | 63.8 | 62.1 |
|  | [70.3,72.7] | [72.0,74.6] | [60.8,63.6] | [62.2,65.4] | [59.3,64.8] |
| Overweight | 15.4 | 11.9 | 15.4 | 13.2 | 13.3 |
|  | [14.5,16.3] | [11.0,12.8] | [14.3,16.5] | [12.1,14.3] | [11.4,15.2] |
| Obese | 12.1 | 13.6 | 21.3 | 20.8 | 22.2 |
|  | [11.4,12.9] | [12.6,14.5] | [20.1,22.6] | [19.5,22.2] | [19.6,24.7] |

Notes: The table shows 95% confidence intervals estimated using the MCS survey design in brackets. The UK90 cut points were used to classify cohort members.

Table S2. Prevalence of obesity over childhood and adolescence by quintiles of permanent family income

| Quintile I |  |  | Age |  |  |
| --- | --- | --- | --- | --- | --- |
|  | 5 | 7 | 11 | 14 | 17 |
| Underweight | 1.2 | 1.6 | 1.5 | 1.7 | 2.6 |
|  | [0.7,1.7] | [0.8,2.4] | [1.0,2.1] | [0.8,2.6] | [1.1,4.1] |
| Normal weight | 73 | 72.4 | 60.7 | 59.3 | 53.5 |
|  | [70.9,75.1] | [69.8,74.9] | [58.2,63.2] | [56.2,62.5] | [47.2,59.8] |
| Overweight | 13.8 | 11.4 | 14.2 | 12.8 | 15.2 |
|  | [12.2,15.5] | [9.8,12.9] | [12.3,16.2] | [10.8,14.7] | [9.7,20.7] |
| Obese | 12 | 14.7 | 23.5 | 26.2 | 28.7 |
|  | [10.5,13.4] | [12.8,16.6] | [21.5,25.6] | [23.2,29.3] | [22.5,34.9] |
| Quintile II |  |  | Age |  |  |
|  | 5 | 7 | 11 | 14 | 17 |
| Underweight | 1 | 1.5 | 1.6 | 1.7 | 2 |
|  | [0.5,1.4] | [0.8,2.1] | [1.0,2.1] | [1.0,2.4] | [1.3,2.8] |
| Normal weight | 71.6 | 71.4 | 60.4 | 58.8 | 57.2 |
|  | [69.6,73.6] | [69.3,73.5] | [57.9,62.9] | [56.4,61.1] | [51.3,63.0] |
| Overweight | 14.6 | 12.4 | 14.4 | 15.7 | 11.3 |
|  | [13.1,16.1] | [10.8,14.1] | [12.6,16.1] | [13.6,17.7] | [9.0,13.6] |
| Obese | 12.9 | 14.7 | 23.7 | 23.9 | 29.5 |
|  | [11.3,14.4] | [13.0,16.4] | [21.6,25.7] | [21.5,26.3] | [24.1,34.9] |
| Quintile III |  |  | Age |  |  |
|  | 5 | 7 | 11 | 14 | 17 |
| Underweight | 0.6 | 0.9 | 1.3 | 1.5 | 2.3 |
|  | [0.3,0.9] | [0.5,1.2] | [0.8,1.7] | [0.7,2.3] | [0.7,3.9] |
| Normal weight | 73.6 | 73 | 59.5 | 60.8 | 59.2 |
|  | [71.9,75.2] | [71.0,75.0] | [57.3,61.8] | [58.4,63.1] | [55.2,63.3] |
| Overweight | 14.5 | 12.8 | 16.9 | 15.2 | 14.9 |
|  | [13.0,15.9] | [11.3,14.2] | [15.0,18.8] | [13.3,17.1] | [12.2,17.6] |
| Obese | 11.4 | 13.4 | 22.3 | 22.5 | 23.5 |
|  | [10.1,12.7] | [11.8,15.0] | [20.2,24.3] | [20.5,24.6] | [19.7,27.4] |
| Quintile IV |  |  | Age |  |  |
|  | 5 | 7 | 11 | 14 | 17 |
| Underweight | 0.7 | 0.8 | 1.4 | 2 | 1.4 |
|  | [0.4,1.0] | [0.5,1.2] | [0.8,1.9] | [1.4,2.6] | [0.9,1.9] |
| Normal weight | 72.8 | 73.8 | 63.2 | 64.8 | 65.5 |
|  | [70.9,74.7] | [72.0,75.6] | [60.8,65.5] | [62.5,67.2] | [62.7,68.3] |
| Overweight | 15.4 | 13.3 | 16.7 | 15.8 | 14.6 |
|  | [14.0,16.9] | [11.9,14.6] | [14.9,18.5] | [14.1,17.6] | [12.5,16.7] |
| Obese | 11.1 | 12.1 | 18.7 | 17.4 | 18.5 |
|  | [9.8,12.4] | [10.8,13.3] | [17.0,20.5] | [15.4,19.3] | [16.3,20.6] |
| Quintile V |  |  | Age |  |  |
|  | 5 | 7 | 11 | 14 | 17 |
| Underweight | 0.6 | 0.8 | 1.5 | 1.3 | 1.5 |
|  | [0.3,0.9] | [0.4,1.1] | [0.9,2.0] | [0.8,1.8] | [0.9,2.1] |
| Normal weight | 77.5 | 79.7 | 71.4 | 72.5 | 70.9 |
|  | [75.9,79.2] | [78.0,81.4] | [69.3,73.5] | [70.4,74.7] | [68.4,73.4] |
| Overweight | 12.5 | 9.8 | 13.5 | 13.2 | 13.7 |
|  | [11.3,13.7] | [8.6,11.0] | [12.1,14.9] | [11.6,14.8] | [11.9,15.5] |
| Obese | 9.4 | 9.7 | 13.6 | 13 | 13.9 |
|  | [8.3,10.5] | [8.4,10.9] | [12.1,15.1] | [11.4,14.5] | [12.1,15.7] |

Notes: The table shows 95% confidence intervals estimated using the MCS survey design in brackets. The UK90 cut points were used to classify cohort members. Statistics are reported by quintiles of family permanent income (see Method section for details).

Table S3. Prevalence of breakfast consumption over childhood and adolescence by quintiles of permanent family income

| Quintile I |  |  | Age |  |  |
| --- | --- | --- | --- | --- | --- |
|  | 5 | 7 | 11 | 14 | 17 |
| Never | 2 | 1.2 | 3.6 | 12 | 16.5 |
|  | [1.4,2.6] | [0.7,1.6] | [2.6,4.5] | [9.9,14.0] | [9.0,24.0] |
| Some days, but not all days | 11.7 | 10.6 | 18.4 | 45.8 | 52.5 |
|  | [10.3,13.1] | [9.2,12.1] | [16.1,20.6] | [42.6,49.1] | [42.6,62.3] |
| Every day | 86.3 | 88.2 | 78.1 | 42.2 | 31.1 |
|  | [84.8,87.8] | [86.7,89.7] | [75.7,80.4] | [38.9,45.5] | [20.3,41.8] |
| Quintile II |  |  | Age |  |  |
|  | 5 | 7 | 11 | 14 | 17 |
| Never | 1.7 | 0.9 | 1.5 | 13.6 | 20.4 |
|  | [1.2,2.3] | [0.4,1.3] | [0.9,2.0] | [11.5,15.7] | [14.3,26.5] |
| Some days, but not all days | 9.7 | 7.5 | 15.9 | 45.9 | 44.3 |
|  | [8.5,11.0] | [6.3,8.6] | [14.1,17.6] | [43.3,48.5] | [37.5,51.1] |
| Every day | 88.5 | 91.7 | 82.7 | 40.5 | 35.3 |
|  | [87.1,89.9] | [90.5,92.9] | [80.9,84.5] | [37.9,43.1] | [28.8,41.8] |
| Quintile III |  |  | Age |  |  |
|  | 5 | 7 | 11 | 14 | 17 |
| Never | 0.5 | 0.3 | 1 | 10.9 | 13.7 |
|  | [0.2,0.8] | [0.1,0.6] | [0.7,1.4] | [9.2,12.7] | [11.0,16.4] |
| Some days, but not all days | 7.5 | 6.5 | 13.3 | 40.1 | 46.9 |
|  | [6.4,8.7] | [5.4,7.5] | [11.6,15.0] | [37.5,42.7] | [43.0,50.7] |
| Every day | 92 | 93.2 | 85.7 | 49 | 39.5 |
|  | [90.8,93.1] | [92.2,94.3] | [83.9,87.4] | [46.4,51.5] | [35.5,43.4] |
| Quintile IV |  |  | Age |  |  |
|  | 5 | 7 | 11 | 14 | 17 |
| Never | 0.4 | 0.5 | 0.9 | 7.8 | 12.4 |
|  | [0.2,0.7] | [0.2,0.8] | [0.5,1.3] | [6.5,9.1] | [10.5,14.4] |
| Some days, but not all days | 4.6 | 3.6 | 10.5 | 35.7 | 40.7 |
|  | [3.7,5.5] | [2.8,4.3] | [9.0,11.9] | [33.5,37.9] | [37.6,43.9] |
| Every day | 95 | 95.9 | 88.6 | 56.5 | 46.8 |
|  | [94.1,95.9] | [95.1,96.7] | [87.2,90.1] | [54.1,58.8] | [43.6,50.1] |
| Quintile V |  |  | Age |  |  |
|  | 5 | 7 | 11 | 14 | 17 |
| Never | 0.4 | 0.1 | 0.4 | 4.4 | 11.5 |
|  | [0.1,0.6] | [-0.0,0.2] | [0.1,0.6] | [3.4,5.5] | [8.4,14.7] |
| Some days, but not all days | 3.2 | 2.4 | 5.5 | 28.5 | 33.8 |
|  | [2.5,3.8] | [1.9,3.0] | [4.5,6.6] | [25.9,31.1] | [30.8,36.7] |
| Every day | 96.5 | 97.5 | 94.1 | 67.1 | 54.7 |
|  | [95.7,97.2] | [97.0,98.1] | [92.9,95.2] | [64.1,70.1] | [51.5,57.9] |

Notes: The table shows 95% confidence intervals estimated using the MCS survey design in brackets. Statistics are reported by quintiles of family permanent income (see Method section for details).

Table S4. Prevalence of physical activity over childhood and adolescence by quintiles of permanent family income

| Quintile I |  |  | Age |  |  |
| --- | --- | --- | --- | --- | --- |
|  | 5 | 7 | 11 | 14 | 17 |
| Not at all | 74.5 | 59.4 | 38.9 | 6.8 | 26.6 |
|  | [72.1,76.9] | [56.4,62.5] | [36.0,41.7] | [4.9,8.8] | [21.0,32.1] |
| 1-4 days | 24.9 | 38.7 | 56.9 | 55.6 | 49.3 |
|  | [22.5,27.4] | [35.6,41.8] | [54.0,59.7] | [52.4,58.8] | [42.7,55.9] |
| 5 or + days | 0.6 | 1.9 | 4.3 | 37.5 | 24.2 |
|  | [0.2,0.9] | [1.1,2.7] | [3.2,5.3] | [34.6,40.5] | [17.8,30.6] |
| Quintile II |  |  | Age |  |  |
|  | 5 | 7 | 11 | 14 | 17 |
| Not at all | 67.9 | 51.9 | 36.5 | 6.9 | 28.5 |
|  | [65.5,70.3] | [49.2,54.5] | [33.8,39.2] | [5.4,8.4] | [23.6,33.3] |
| 1-4 days | 31.6 | 46.9 | 59.1 | 59.5 | 46.1 |
|  | [29.2,33.9] | [44.2,49.6] | [56.5,61.6] | [56.8,62.3] | [40.8,51.4] |
| 5 or + days | 0.5 | 1.2 | 4.4 | 33.6 | 25.5 |
|  | [0.2,0.8] | [0.7,1.7] | [3.3,5.4] | [30.8,36.4] | [18.1,32.8] |
| Quintile III |  |  | Age |  |  |
|  | 5 | 7 | 11 | 14 | 17 |
| Not at all | 50.1 | 33.5 | 29 | 4.9 | 24.2 |
|  | [47.6,52.6] | [31.4,35.6] | [26.7,31.4] | [3.7,6.1] | [20.8,27.6] |
| 1-4 days | 49.2 | 64.4 | 64.1 | 57.9 | 52.3 |
|  | [46.7,51.7] | [62.3,66.6] | [61.7,66.4] | [55.1,60.7] | [48.2,56.5] |
| 5 or + days | 0.7 | 2.1 | 6.9 | 37.2 | 23.4 |
|  | [0.4,1.0] | [1.4,2.7] | [5.8,8.1] | [34.3,40.1] | [19.7,27.2] |
| Quintile IV |  |  | Age |  |  |
|  | 5 | 7 | 11 | 14 | 17 |
| Not at all | 35 | 20.8 | 21.1 | 4.2 | 22.3 |
|  | [33.0,37.1] | [19.0,22.7] | [19.1,23.1] | [3.2,5.2] | [18.4,26.1] |
| 1-4 days | 63.9 | 76.4 | 69.7 | 58.5 | 56.9 |
|  | [61.8,65.9] | [74.7,78.1] | [67.5,71.9] | [56.1,60.9] | [52.9,60.9] |
| 5 or + days | 1.1 | 2.8 | 9.1 | 37.3 | 20.8 |
|  | [0.7,1.5] | [2.2,3.4] | [7.8,10.5] | [35.0,39.6] | [18.4,23.2] |
| Quintile V |  |  | Age |  |  |
|  | 5 | 7 | 11 | 14 | 17 |
| Not at all | 22.2 | 10.7 | 13.1 | 2.3 | 16.4 |
|  | [20.3,24.1] | [9.3,12.0] | [11.6,14.7] | [1.6,2.9] | [14.4,18.4] |
| 1-4 days | 76.8 | 85 | 72.8 | 54.3 | 58.7 |
|  | [74.9,78.7] | [83.6,86.3] | [70.8,74.8] | [52.0,56.6] | [56.1,61.3] |
| 5 or + days | 1 | 4.4 | 14.1 | 43.4 | 24.9 |
|  | [0.6,1.4] | [3.6,5.2] | [12.2,16.1] | [41.1,45.8] | [22.6,27.2] |

Notes: The table shows 95% confidence intervals estimated using the MCS survey design in brackets. Statistics are reported by quintiles of family permanent income (see Method section for details).

Table S5. Descriptive statistics of cohort members eating behaviours at ages 11, 14 and 17, by breakfast consumption at age 7.

|  | Days per week eats breakfast | | | | | |
| --- | --- | --- | --- | --- | --- | --- |
|  | Never or some days | | | Every day | | |
|  | 11 | 14 | 17 | 11 | 14 | 17 |
| How often does cohort member drink sweetened drinks? |  |  |  |  |  |  |
| *More than once a day* | 21.0 | 17.2 | 15.3 | 14.2 | 10.9 | 7.2 |
| *Once a day* | 20.3 | 21.1 | 17.4 | 16.2 | 13.0 | 9.6 |
| *3-6 days a week* | 11.6 | 20.4 | 14.9 | 8.6 | 20.8 | 15.4 |
| *1-2 days a week* | 21.2 | 21.6 | 19.6 | 20.2 | 24.5 | 24.0 |
| *Less often but at least once a month* | 8.3 | 7.1 | 10.3 | 12.4 | 13.6 | 16.8 |
| *Less than once a month* | 5.8 | 2.5 | 6.9 | 9.5 | 6.2 | 8.5 |
| *Hardly ever or never* | 11.7 | 10.2 | 15.5 | 18.9 | 11.0 | 18.5 |
| How often does cohort member eat fast food? |  |  |  |  |  |  |
| *More than once a day* |  | 2.2 | 0.9 |  | 0.7 | 0.4 |
| *Once a day* |  | 1.9 | 0.7 |  | 0.9 | 0.5 |
| *3-6 days a week* |  | 8.4 | 10.9 |  | 4.3 | 4.8 |
| *1-2 days a week* |  | 30.7 | 35.6 |  | 21.1 | 24.2 |
| *Less often but at least once a month* |  | 39.6 | 32.2 |  | 45.1 | 41.6 |
| *Less than once a month* |  | 13.5 | 13.4 |  | 23.5 | 18.0 |
| *Hardly ever or never* |  | 3.8 | 6.2 |  | 4.4 | 10.5 |
| How often does cohort member eat at least 2 portions of vegetables? |  |  |  |  |  |  |
| *Never* |  | 14.1 | 12.1 |  | 7.5 | 6.7 |
| *Some days, but not all days* |  | 63.7 | 61.0 |  | 54.6 | 49.6 |
| *Every day* |  | 22.1 | 26.8 |  | 38.0 | 43.7 |

Notes: First three columns indicate cohort member ages from 11 to 17 among those who declared eating breakfast irregularly at age 7 (categories ‘never’ and ‘some days’), and the last three columns among those who declared eating breakfast regularly at age 7 (‘every day’).

Table S6. Estimate of Eating Choice Index (ECI) mean by cohort member’s age and breakfast consumption at age 7.

| Age | Days per week eats breakfast | Mean | Std. Err. | [95% Conf. Interval] | |
| --- | --- | --- | --- | --- | --- |
| 14 | Never or some days | 10.6 | 0.2 | 10.2 | 11.0 |
|  | Every day | 12.1 | 0.1 | 12.0 | 12.2 |
| 17 | Never or some days | 10.8 | 0.2 | 10.4 | 11.2 |
|  | Every day | 12.3 | 0.1 | 12.2 | 12.4 |

Notes: The Eating Choice Index (ECI) discriminates healthy and unhealthy eating choices, where higher scores indicate healthy eating behaviours. It includes four components: i) consumption of breakfast ii) consumption of two portion of fruit per day, iii) type of milk consumed and iv) type of bread consumed, each providing a score from 1 to 5. At ages 17 and 14, we reject the null of equal ECI means by cohort member’s breakfast consumption at age 7, indicating that children who eat breakfast regularly, show healthy eating choices during adolescence. MCS sampling design and cross-sectional weights were used standard errors.

Figure S1. Eating Choice Index distribution by cohort member’s age and breakfast consumption at age 7.


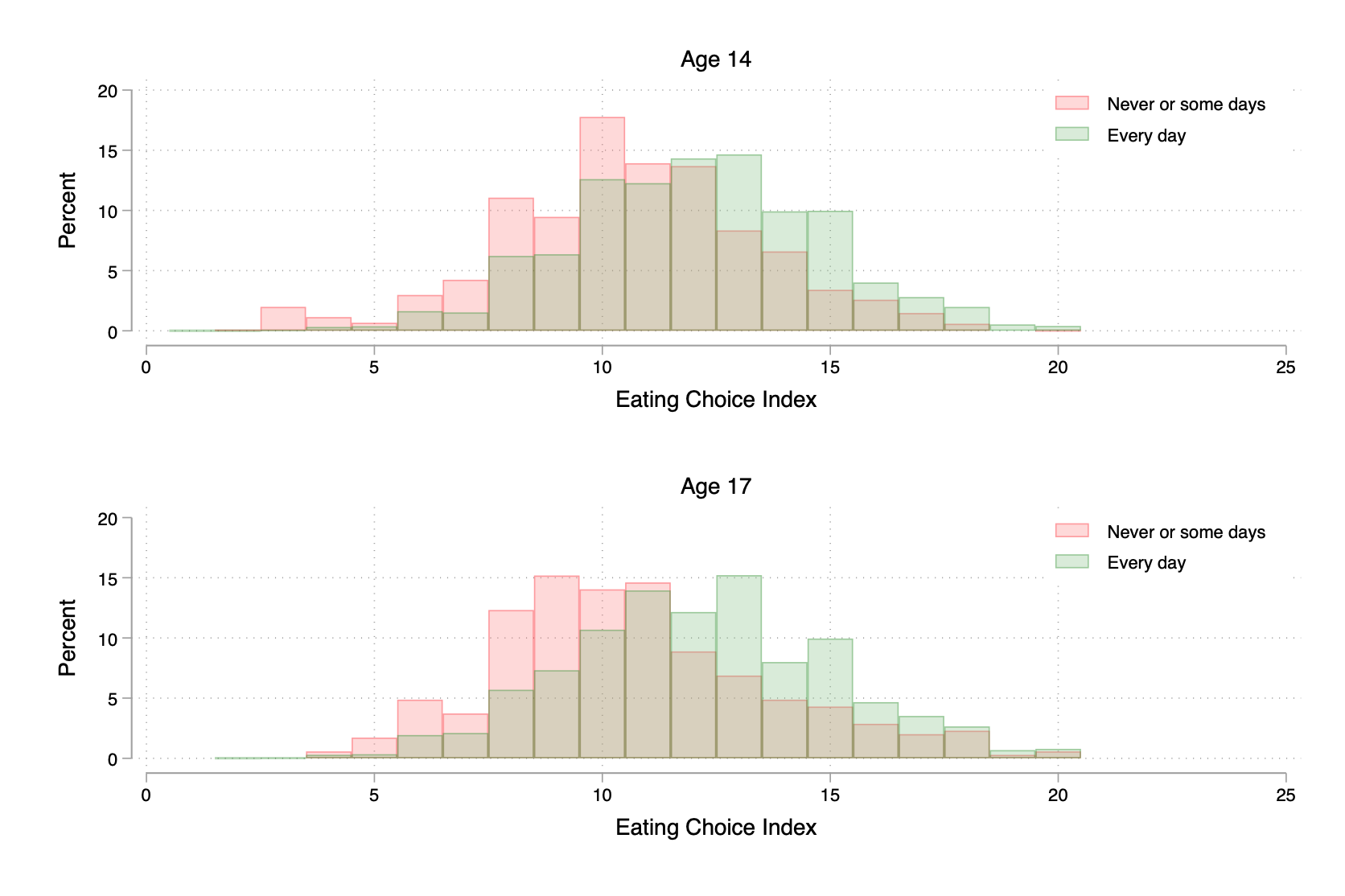


Notes: This figure shows the Eating Choice Index distribution by cohort member’s age and breakfast consumption at age 7. It shows the positive association between eating breakfast regularly during childhood and healthy eating behaviours during adolescence.

Table S7. Prevalence of obesity over childhood and adolescence (IOTF)

|  |  |  | All |  |  |
| --- | --- | --- | --- | --- | --- |
|  | 5 | 7 | 11 | 14 | 17 |
| Underweight | 4.1 | 5.9 | 6.2 | 6.6 | 9.1 |
|  | [3.6,4.5] | [5.4,6.4] | [5.7,6.7] | [6.0,7.2] | [7.7,10.5] |
| Normal weight | 75.1 | 73.9 | 66.3 | 66.2 | 61.2 |
|  | [74.3,75.9] | [73.0,74.8] | [65.4,67.3] | [65.1,67.3] | [59.4,63.0] |
| Overweight | 15.4 | 14.5 | 21 | 19.2 | 19 |
|  | [14.7,16.1] | [13.9,15.2] | [20.2,21.9] | [18.2,20.1] | [17.5,20.4] |
| Obese | 5.4 | 5.7 | 6.5 | 8 | 10.7 |
|  | [5.0,5.9] | [5.2,6.2] | [5.9,7.0] | [7.3,8.7] | [9.3,12.2] |
|  |  |  | Female |  |  |
|  | 5 | 7 | 11 | 14 | 17 |
| Underweight | 3.7 | 5.9 | 7 | 5.9 | 7.7 |
|  | [3.2,4.2] | [5.2,6.6] | [6.2,7.8] | [5.1,6.7] | [6.7,8.8] |
| Normal weight | 73.2 | 71.2 | 63.4 | 65.5 | 61.1 |
|  | [72.0,74.3] | [69.9,72.5] | [61.9,64.9] | [63.8,67.2] | [58.9,63.4] |
| Overweight | 17.3 | 16.3 | 23 | 20.4 | 20.1 |
|  | [16.3,18.4] | [15.3,17.4] | [21.7,24.3] | [18.9,21.8] | [18.3,21.9] |
| Obese | 5.8 | 6.5 | 6.6 | 8.3 | 11.1 |
|  | [5.1,6.4] | [5.8,7.3] | [5.9,7.4] | [7.3,9.3] | [9.1,13.0] |
|  |  |  | Male |  |  |
|  | 5 | 7 | 11 | 14 | 17 |
| Underweight | 4.4 | 5.9 | 5.4 | 7.3 | 10.4 |
|  | [3.8,5.0] | [5.2,6.5] | [4.8,6.0] | [6.4,8.1] | [7.8,13.0] |
| Normal weight | 76.9 | 76.5 | 69.1 | 66.8 | 61.3 |
|  | [75.7,78.1] | [75.3,77.6] | [67.8,70.4] | [65.2,68.4] | [58.4,64.2] |
| Overweight | 13.5 | 12.8 | 19.2 | 18.1 | 17.9 |
|  | [12.6,14.5] | [11.9,13.7] | [18.0,20.4] | [16.7,19.5] | [15.6,20.2] |
| Obese | 5.1 | 4.9 | 6.3 | 7.8 | 10.4 |
|  | [4.6,5.7] | [4.3,5.5] | [5.6,7.0] | [6.9,8.7] | [8.4,12.4] |

Notes: The table shows 95% confidence intervals estimated using the MCS survey design in brackets. The IOTF cut points were used to classify cohort members.

Table S8. Prevalence of obesity over childhood and adolescence by quintiles of permanent family income (IOTF)

|  |  |  | I |  |  |
| --- | --- | --- | --- | --- | --- |
|  | 5 | 7 | 11 | 14 | 17 |
| Underweight | 6.2 | 7.4 | 7.3 | 7 | 8.6 |
|  | [4.9,7.4] | [6.0,8.8] | [6.0,8.7] | [5.4,8.6] | [6.0,11.3] |
| Normal weight | 72.7 | 70.3 | 62.9 | 60.8 | 55.2 |
|  | [70.7,74.7] | [68.0,72.6] | [60.4,65.4] | [57.5,64.0] | [49.1,61.2] |
| Overweight | 14.4 | 14.7 | 21.1 | 20.4 | 17.2 |
|  | [12.7,16.0] | [13.2,16.3] | [19.1,23.2] | [17.7,23.0] | [13.1,21.2] |
| Obese | 6.8 | 7.6 | 8.6 | 11.9 | 19 |
|  | [5.7,7.8] | [6.4,8.8] | [7.2,10.1] | [10.0,13.8] | [12.9,25.2] |
|  |  |  | II |  |  |
|  | 5 | 7 | 11 | 14 | 17 |
| Underweight | 4.4 | 6.3 | 5.9 | 6.9 | 12.1 |
|  | [3.5,5.3] | [5.1,7.5] | [4.7,7.0] | [5.2,8.7] | [5.0,19.3] |
| Normal weight | 72.5 | 70.9 | 63 | 61.7 | 50.7 |
|  | [70.5,74.5] | [68.5,73.2] | [60.5,65.4] | [58.9,64.5] | [44.6,56.7] |
| Overweight | 16.2 | 15.9 | 22.7 | 21 | 22.4 |
|  | [14.5,17.8] | [14.3,17.5] | [20.6,24.8] | [18.6,23.3] | [17.6,27.3] |
| Obese | 6.9 | 7 | 8.5 | 10.3 | 14.8 |
|  | [5.7,8.1] | [5.7,8.3] | [7.0,9.9] | [8.5,12.2] | [10.6,19.0] |
|  |  |  | III |  |  |
|  | 5 | 7 | 11 | 14 | 17 |
| Underweight | 3 | 5 | 5.5 | 6 | 8.5 |
|  | [2.3,3.7] | [4.2,5.9] | [4.5,6.5] | [4.8,7.3] | [6.4,10.6] |
| Normal weight | 75.6 | 73.5 | 63.8 | 64.3 | 59.3 |
|  | [73.9,77.3] | [71.5,75.4] | [61.7,65.9] | [61.9,66.7] | [55.5,63.1] |
| Overweight | 16.1 | 15.5 | 23.6 | 21.3 | 21.8 |
|  | [14.6,17.6] | [14.0,17.0] | [21.7,25.4] | [19.0,23.6] | [17.7,25.8] |
| Obese | 5.3 | 6 | 7.1 | 8.4 | 10.4 |
|  | [4.3,6.3] | [5.0,7.0] | [5.9,8.4] | [7.0,9.8] | [8.5,12.3] |
|  |  |  | IV |  |  |
|  | 5 | 7 | 11 | 14 | 17 |
| Underweight | 3.4 | 5.3 | 5.3 | 5.3 | 7.9 |
|  | [2.7,4.2] | [4.4,6.3] | [4.4,6.2] | [4.4,6.2] | [5.9,10.0] |
| Normal weight | 74.6 | 74.5 | 67.6 | 70.4 | 64.5 |
|  | [72.8,76.4] | [72.7,76.2] | [65.6,69.6] | [68.3,72.6] | [61.9,67.2] |
| Overweight | 17.1 | 15.5 | 21.8 | 18.7 | 19.2 |
|  | [15.5,18.8] | [14.2,16.9] | [20.0,23.6] | [17.0,20.4] | [17.1,21.3] |
| Obese | 4.8 | 4.7 | 5.3 | 5.6 | 8.4 |
|  | [4.0,5.7] | [3.9,5.5] | [4.3,6.3] | [4.3,6.8] | [6.7,10.1] |
|  |  |  | V |  |  |
|  | 5 | 7 | 11 | 14 | 17 |
| Underweight | 4 | 5.8 | 7 | 7.9 | 9 |
|  | [3.3,4.7] | [4.8,6.7] | [6.0,7.9] | [6.7,9.0] | [7.6,10.3] |
| Normal weight | 78.5 | 78.6 | 73.4 | 73.7 | 69.4 |
|  | [76.7,80.2] | [77.0,80.3] | [71.4,75.4] | [71.8,75.7] | [66.9,72.0] |
| Overweight | 13.3 | 11.5 | 16.4 | 14.4 | 16 |
|  | [12.0,14.6] | [10.4,12.7] | [14.9,17.8] | [12.8,16.1] | [14.3,17.7] |
| Obese | 4.2 | 4.1 | 3.2 | 3.9 | 5.6 |
|  | [3.4,5.1] | [3.2,4.9] | [2.4,4.1] | [3.1,4.8] | [4.5,6.7] |

Notes: The table shows 95% confidence intervals estimated using the MCS survey design in brackets. The IOTF cut points were used to classify cohort members.

Table S9. Prevalence of breakfast consumption and physical activity

|  | Age | | | | |
| --- | --- | --- | --- | --- | --- |
|  | 5 | 7 | 11 | 14 | 17 |
| Not at all | 46.8 [45.0,48.6] | 33.2 [31.4,35.0] | 27.4 [25.9,28.8] | 5.1 [4.4,5.7] | 23.2 [21.4,25.0] |
| 1-4 days | 52.4 [50.6,54.2] | 64.2 [62.5,65.9] | 64.7 [63.5,65.9] | 57.2 [55.9,58.5] | 53.2 [51.2,55.2] |
| 5 or + days | 0.8 [0.6,1.0] | 2.6 [2.2,2.9] | 7.9 [7.1,8.7] | 37.8 [36.5,39.0] | 23.6 [21.6,25.6] |

Notes: The table shows 95% confidence intervals estimated using the MCS survey design in brackets.

Table S10. Prevalence of physical activity

|  | Age | | | | |
| --- | --- | --- | --- | --- | --- |
|  | 5 | 7 | 11 | 14 | 17 |
| Never | 0.9 [0.7,1.1] | 0.5 [0.4,0.7] | 1.4 [1.2,1.7] | 9.8 [9.1,10.6] | 14.5 [12.6,16.4] |
| Some days, but not all days | 6.8 [6.4,7.3] | 5.8 [5.3,6.2] | 12.5 [11.7,13.3] | 39.3 [38.1,40.6] | 41.7 [39.5,43.9] |
| Every day | 92.3 [91.7,92.8] | 93.7 [93.2,94.2] | 86.1 [85.2,86.9] | 50.9 [49.5,52.2] | 43.8 [41.5,46.2] |

Notes: The table shows 95% confidence intervals estimated using the MCS survey design in brackets.

Table S11. Multilevel linear regression models

|  | (1) | | (2) | | (3) | |
| --- | --- | --- | --- | --- | --- | --- |
|  | z-BMI | | BMI | | Body fat (%) | |
| Ridit score (permanent income) | 0.00^*^ | [-0.00,0.00] | 0.01^***^ | [0.00,0.01] | -0.00 | [-0.01,0.01] |
| Age at interview | 0.03^***^ | [0.03,0.04] | 0.68^***^ | [0.66,0.70] | 0.23^***^ | [0.18,0.27] |
| Ridit score (permanent income) # Age at interview | -0.00^***^ | [-0.00,-0.00] | -0.00^***^ | [-0.00,-0.00] | -0.00^***^ | [-0.00,-0.00] |
| Sex |  |  |  |  |  |  |
| Female |  |  | Ref. |  | Ref. |  |
| Male |  |  | 0.09^*^ | [-0.00,0.18] | -3.67^***^ | [-3.96,-3.38] |
| Constant | 0.18^***^ | [0.10,0.26] | 12.14^***^ | [12.01,12.27] | 21.97^***^ | [21.45,22.49] |
| sd(Age) | 0.07^***^ | [0.06,0.07] | 0.28^***^ | [0.27,0.29] | 0.59^***^ | [0.55,0.63] |
| sd(_cons) | 0.95^**^ | [0.91,0.99] | 1.15^*^ | [0.99,1.34] | 4.92^***^ | [4.18,5.80] |
| sd(Residual) | -0.42^***^ | [-0.49,-0.36] | -0.62^***^ | [-0.69,-0.54] | -0.80^***^ | [-0.90,-0.71] |
| lnsig_e | 0.54^***^ | [0.51,0.56] | 1.52^***^ | [1.46,1.59] | 4.10^***^ | [3.88,4.34] |
| Observations | 36100 |  | 36100 |  | 28554 |  |

Notes: We report 95% confidence intervals in brackets. ***, ** and * denote statistical significance at 1%, 5% and 10% levels respectively. Confidence intervals are calculated using MCS survey design and estimates are calculated using survey weight to account for attrition at age 17.

Table S12. Multilevel linear regression models

|  | (1) | | (2) | |
| --- | --- | --- | --- | --- |
|  | Weekly physical activity  (5 + days) | | Eats breakfast every day of week | |
| Ridit score (permanent income) | 0.00 | [-0.00,0.00] | -0.00^**^ | [-0.00,-0.00] |
| Age at interview | 0.02^***^ | [0.02,0.02] | -0.05^***^ | [-0.06,-0.05] |
| Ridit score (permanent income) # Age at interview | 0.00^***^ | [0.00,0.00] | 0.00^***^ | [0.00,0.00] |
| Constant | -0.14^***^ | [-0.16,-0.12] | 1.26^***^ | [1.24,1.28] |
| sd(Age) | 0.02^***^ | [0.02,0.02] | 0.03^***^ | [0.03,0.03] |
| sd(_cons) | 0.13^***^ | [0.12,0.15] | 0.17^***^ | [0.16,0.18] |
| sd(Residual) | -10.76 | [-830.58,809.07] | -10.23 | [-665.12,644.65] |
| lnsig_e | 0.30^***^ | [0.30,0.30] | 0.30^***^ | [0.30,0.30] |
| Observations | 36020 |  | 33855 |  |

Notes: We report 95% confidence intervals in brackets. ***, ** and * denote statistical significance at 1%, 5% and 10% levels respectively. Confidence intervals are calculated using MCS survey design and estimates are calculated using survey weight to account for attrition at age 17.

Table S13. Descriptive statistics of FE sample and excluded sample (n=346) due to missing covariates

|  | FE sample | | Sample with missing covariates | |
| --- | --- | --- | --- | --- |
|  | % | 95% CI | % | 95% CI |
| Female | 50.9 | [49.5,52.4] | 48.0 | [41.8,54.3] |
| Ethnicity |  |  |  |  |
| White | 87.0 | [84.5,89.1] | 83.3 | [76.7,88.3] |
| Mixed | 2.8 | [2.3,3.3] | 4.8 | [2.5,8.9] |
| Indian | 2.2 | [1.6,2.9] | 1.9 | [0.9,4.1] |
| Pakistani and Bangladeshi | 4.2 | [2.8,6.2] | 6.6 | [3.4,12.3] |
| Black or Black British | 2.2 | [1.5,3.3] | 1.5 | [0.6,3.6] |
| Other Ethnic group | 1.7 | [1.3,2.2] | 2.0 | [0.8,4.4] |
| Urban | 77.4 | [73.7,80.8] | 75.6 | [67.3,82.4] |
| Region |  |  |  |  |
| North East | 3.0 | [1.7,5.5] | 0.8 | [0.2,2.4] |
| North West | 9.3 | [6.1,14.0] | 16.2 | [9.2,26.9] |
| Yorkshire and the Humber | 8.8 | [5.1,14.8] | 9.3 | [4.0,20.3] |
| East Midlands | 8.0 | [5.2,12.1] | 6.1 | [3.3,11.1] |
| West Midlands | 7.5 | [4.6,12.0] | 6.5 | [3.2,12.7] |
| East of England | 10.0 | [6.9,14.4] | 13.4 | [7.8,22.3] |
| London | 11.3 | [7.7,16.3] | 13.0 | [6.3,24.7] |
| South East | 16.1 | [11.8,21.6] | 17.0 | [9.8,27.9] |
| South West | 9.5 | [6.2,14.2] | 4.1 | [2.0,8.3] |
| Wales | 4.9 | [4.0,5.9] | 4.3 | [2.8,6.5] |
| Scotland | 7.9 | [6.9,9.0] | 6.2 | [4.2,9.1] |
| Northern Ireland | 3.7 | [3.2,4.3] | 3.1 | [2.0,4.8] |
| Combined labour market status |  |  |  |  |
| Both in work | 53.9 | [52.0,55.7] | 57.9 | [51.4,64.1] |
| Only one in work, (main or partner) | 28.1 | [26.6,29.8] | 30.2 | [25.0,35.9] |
| Both not in work | 3.8 | [3.2,4.4] | 4.9 | [2.9,8.1] |
| Main in work or on leave, no partner | 7.1 | [6.3,7.9] | 3.7 | [1.9,6.9] |
| Main not in work nor on leave, no partner | 7.1 | [6.4,8.0] | 3.4 | [1.9,6.1] |
| Number of siblings in household plus cohort member | 1.4 | [1.4,1.5] | 1.2 | [1.1,1.4] |
| Permanent OECD equivalised weekly family income (0-5y) | 356.0 | [341.3,370.7] | 483.9 | [424.8,543.1] |
| Observations | 6883 |  | 346 |  |

Notes: Variables are recorded at age 5. We report 95% estimates and confidence intervals weighted to considering sampling weights at age the sweep age 5 and MCS survey design.

Table S14. Association between demographic characteristics and excluded participants due to missing on covariates

|  | Odd Ratios | 95% CI |
| --- | --- | --- |
|  |  |  |
| Sex |  |  |
| Female | 0.894 | [0.688,1.162] |
| Male | Ref. |  |
| Ethnicity |  |  |
| White | Ref. |  |
| Mixed | 1.855^*^ | [0.965,3.564] |
| Indian | 0.991 | [0.424,2.318] |
| Pakistani and Bangladeshi | 2.844^***^ | [1.645,4.919] |
| Black or Black British | 1.105 | [0.383,3.189] |
| Other Ethnic group | 1.690 | [0.670,4.261] |
| Urban | Ref. |  |
| Rural | 1.108 | [0.756,1.623] |
| Region |  |  |
| North East | 0.323 | [0.0802,1.303] |
| North West | 2.122^**^ | [1.106,4.072] |
| Yorkshire and the Humber | 1.388 | [0.667,2.887] |
| East Midlands | 0.955 | [0.463,1.969] |
| West Midlands | 1.082 | [0.524,2.234] |
| East of England | 1.374 | [0.703,2.685] |
| London | Ref. |  |
| South East | 1.049 | [0.486,2.263] |
| South West | 0.559 | [0.239,1.307] |
| Wales | 1.219 | [0.610,2.435] |
| Scotland | 0.924 | [0.471,1.811] |
| Northern Ireland | 1.336 | [0.659,2.708] |
| Combined labour market status |  |  |
| Both in work | Ref. |  |
| Only one in work, (main or partner) | 1.277 | [0.946,1.724] |
| Both not in work | 2.969^***^ | [1.563,5.639] |
| Main in work or on leave, no partner | 0.659 | [0.333,1.305] |
| Main not in work nor on leave, no partner | 1.097 | [0.576,2.091] |
| Number of siblings in household plus cohort member | 0.719^***^ | [0.579,0.893] |
| Permanent OECD equivalised weekly family income (0-5y) | 1.003^***^ | [1.002,1.004] |
| Observations | 7229 |  |

Notes: This table show results from a Logistic model with a dependent variable defined as 1 if there is a missing observation on covariates used in the FE models. We report 95% confidence intervals in brackets. ***, ** and * denote statistical significance at 1%, 5% and 10% levels respectively. OLS estimates show associations with BMI and Body fat percentage.

Table S15. FE estimates using multi-imputation for missing covariates

|  | BMI | | Body fat (%) | |
| --- | --- | --- | --- | --- |
|  | FE | 95% CI | FE | 95% CI |
| Days per week eats breakfast ^a^ |  |  |  |  |
| Never (never) | Ref |  | Ref |  |
| Some days, but not all days (irregular) | -0.05 | [-0.36,0.25] | -0.62* | [-1.25,0.01] |
| Every day (regular) | -0.17 | [-0.47,0.14] | -1.45*** | [-2.08,-0.82] |
| Weekly physical activity ^a^ |  |  |  |  |
| Not at all (never) | Ref |  | Ref |  |
| 1-4 days (irregular) | -0.02 | [-0.11,0.07] | -0.43*** | [-0.65,-0.22] |
| 5 or + days (regular) | -0.14* | [-0.27,0.00] | -1.03*** | [-1.37,-0.70] |
| Log of deflated OECD equivalised weekly family income ^a^ | 0.12** | [0.01,0.22] | 0.17 | [-0.11,0.45] |
| Number of siblings in household plus cohort member | -0.10** | [-0.18,-0.02] | -0.17* | [-0.36,0.02] |
| Housing Tenure ^a^ |  |  |  |  |
| Rent house or other | Ref |  | Ref |  |
| Own house | -0.19* | [-0.39,0.01] | -0.43* | [-0.90,0.03] |
| Combined labour market status ^a^ |  |  |  |  |
| Both in work | Ref |  | Ref |  |
| Only one in work, (main or partner) | 0.03 | [-0.08,0.14] | -0.02 | [-0.30,0.25] |
| Both not in work | -0.07 | [-0.33,0.20] | -0.23 | [-0.88,0.43] |
| Main in work or on leave, no partner | 0.14 | [-0.04,0.31] | 0.19 | [-0.25,0.62] |
| Main not in work nor on leave, no partner | 0.12 | [-0.12,0.36] | -0.22 | [-0.79,0.35] |
| Sweeps |  |  |  |  |
| Sweep=7 | Ref |  | Ref |  |
| Sweep=11 | 0.03 | [-0.88,0.93] | 1.55 | [-0.74,3.84] |
| Sweep=14 | 0.53 | [-0.89,1.94] | 3.61** | [0.02,7.20] |
| Sweep=17 | 1.06 | [-0.78,2.90] | 7.99*** | [3.36,12.62] |
| Age at interview | 0.95*** | [0.47,1.42] | 1.83*** | [0.64,3.02] |
| Age at interview squared | -0.02** | [-0.03,-0.00] | -0.10*** | [-0.14,-0.06] |
| Unemployment rate (LA) | -0.05*** | [-0.08,-0.02] | -0.06 | [-0.13,0.02] |
| Constant | 10.50*** | [7.74,13.26] | 14.33*** | [7.41,21.25] |
| Observations | 28746 |  | 28423 |  |

Notes: This table reports FE estimates using multiple-imputation methods with 10 imputed datasets. Missing values of covariates were imputed using chained equations. An order Logit model was used to impute days per week eats breakfast and weekly physical activity; a Logit model was used to impute housing tenure; a Poisson model was used to impute the number of siblings in household plus cohort member; and a linear regression was used to impute the log of deflated OECD equivalised weekly family income. The covariates used in the imputation models were sex, age at interview, age at interview squared, combined labour market status, ethnicity, rural status, and region. FE confidence intervals are calculated using cluster standard error at individual level. FE estimates are calculated using survey weight to account for attrition at age 17. We report 95% confidence intervals in brackets. ***, ** and * denote statistical significance at 1%, 5% and 10% levels respectively. OLS estimates show associations with BMI and Body fat percentage. FE estimates show associations with changes over ages 7-17. ^a^ Indicates the lag value of the variable.

Figure S2. Directed acyclic graph (DAG)


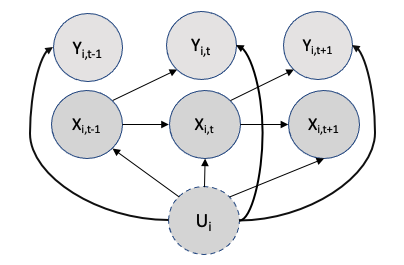


Notes: This figure illustrates the identification assumptions in a linear fixed effect model with one treatment variable. Solid circles represent observed outcome Yi,t and treatment Xi,t-1 variables. A dashed circle represents a vector of unobserved, unit-specific and time-invariant confounders (Ui). Yit represents BMI, and Xi,t-1 is lagged behaviours. The absence of arrows represents the lack of causal relationship. For simplicity, we include one treatment and three periods, but more detailed DAG structures can be found in Imai & Kim (2019). The assumption in this DAG are (a) No unobserved time-varying confounder exists. (b) Past outcomes do not directly affect current outcome. (c) Past outcomes do not directly affect current treatment. (d) Current treatments do not directly affect current outcome.

Imai K, Kim IS. When Should We Use Unit Fixed Effects Regression Models for Causal Inference with Longitudinal Data? American Journal of Political Science. 2019;63(2):467-90.

Table S16. FE estimates, additional model specifications

|  | BMI | | | | Body fat (%) | | | |
| --- | --- | --- | --- | --- | --- | --- | --- | --- |
|  | Model 1 | | Model 2 | | Model 1 | | Model 2 | |
|  | FE | 95% CI | FE | 95% CI | FE | 95% CI | FE | 95% CI |
| BMI (previous survey) |  |  | 0.44*** | [0.41,0.47] |  |  |  |  |
| Body fat (previous survey) |  |  |  |  |  |  | 0.12*** | [0.10,0.14] |
| Days per week eats breakfast (current) |  |  |  |  |  |  |  |  |
| Never (never) | Ref. |  | Ref. |  | Ref. |  | Ref. |  |
| Some days, but not all days (irregular) | -0.20** | [-0.38,-0.03] | -0.06 | [-0.21,0.10] | -0.19 | [-0.61,0.22] | -0.13 | [-0.55,0.29] |
| Every day (regular) | -0.48*** | [-0.66,-0.30] | -0.21** | [-0.36,-0.05] | -0.65*** | [-1.08,-0.21] | -0.54** | [-0.97,-0.11] |
| Weekly physical activity (current) |  |  |  |  |  |  |  |  |
| Not at all (never) | Ref. |  | Ref. |  | Ref. |  | Ref. |  |
| 1-4 days (irregular) | 0.04 | [-0.06,0.14] | 0.01 | [-0.08,0.09] | -0.53*** | [-0.81,-0.25] | -0.53*** | [-0.81,-0.26] |
| 5 or + days (regular) | -0.27*** | [-0.39,-0.14] | -0.24*** | [-0.35,-0.12] | -1.43*** | [-1.78,-1.08] | -1.39*** | [-1.73,-1.04] |
| Log of deflated OECD equivalised weekly family income ^a^ | 0.12** | [0.01,0.23] | 0.05 | [-0.04,0.15] | -0.05 | [-0.41,0.31] | -0.10 | [-0.45,0.25] |
| Number of siblings in household plus cohort member | -0.09** | [-0.18,-0.01] | -0.05 | [-0.12,0.01] | 0.04 | [-0.21,0.29] | 0.06 | [-0.18,0.31] |
| Housing Tenure ^a^ |  |  |  |  |  |  |  |  |
| Rent house or other | Ref. |  | Ref. |  | Ref. |  | Ref. |  |
| Own house | -0.19* | [-0.39,0.02] | -0.08 | [-0.25,0.09] | -0.16 | [-0.73,0.41] | -0.09 | [-0.66,0.48] |
| Combined labour market status ^a^ |  |  |  |  |  |  |  |  |
| Both in work | Ref. |  | Ref. |  | Ref. |  | Ref. |  |
| Only one in work, (main or partner) | 0.04 | [-0.07,0.16] | -0.01 | [-0.11,0.09] | -0.23 | [-0.56,0.11] | -0.2 | [-0.53,0.13] |
| Both not in work | -0.06 | [-0.34,0.21] | -0.02 | [-0.26,0.23] | -0.69* | [-1.45,0.07] | -0.58 | [-1.34,0.18] |
| Main in work or on leave, no partner | 0.13 | [-0.04,0.31] | 0.05 | [-0.10,0.20] | 0.11 | [-0.36,0.58] | 0.07 | [-0.41,0.54] |
| Main not in work nor on leave, no partner | 0.16 | [-0.09,0.40] | 0.05 | [-0.15,0.26] | -0.20 | [-0.88,0.48] | -0.21 | [-0.88,0.45] |
| Sweeps |  |  |  |  |  |  |  |  |
| Sweep=7 | Ref. |  | Ref. |  |  |  |  |  |
| Sweep=11 | 0.30 | [-0.62,1.22] | -0.38 | [-1.19,0.43] | Ref. |  | Ref. |  |
| Sweep=14 | 1.01 | [-0.43,2.45] | -0.88 | [-2.18,0.42] | 3.12*** | [1.40,4.83] | 3.23*** | [1.51,4.95] |
| Sweep=17 | 1.63* | [-0.23,3.48] | -1.30 | [-2.99,0.40] | 6.79*** | [3.69,9.89] | 6.92*** | [3.80,10.04] |
| Age at interview | 0.85*** | [0.37,1.34] | 1.15*** | [0.76,1.54] | -0.17 | [-1.72,1.39] | -0.60 | [-2.10,0.91] |
| Age at interview squared | -0.02* | [-0.03,0.00] | -0.02*** | [-0.04,-0.01] | -0.04 | [-0.09,0.02] | -0.02 | [-0.07,0.03] |
| Unemployment rate (LA) | -0.05*** | [-0.08,-0.02] | -0.02 | [-0.04,0.01] | -0.01 | [-0.11,0.08] | 0.00 | [-0.09,0.09] |
| Constant | 11.33*** | [8.52,14.14] | 2.36** | [0.01,4.71] | 29.41*** | [17.53,41.29] | 29.94*** | [18.36,41.52] |
| Observations | 27411 |  | 27411 |  | 20347 |  | 20151 |  |

Note: This table shows FE estimates relaxing third and fourth assumptions as described in the Method section. Model 1 relax the assumption that current behaviours do not affect current BMI/body fat by including current breakfast consumption and physical activity. Model 2 relax the assumptions that past BMI/body fat does not directly affect current BMI/body fat by adding the lag of outcomes to model 1. Because 30% of breakfast consumption at age 17 was missing in the analytical sample, we used multiple imputation techniques for this variable to perform this robustness exercise. We use five imputations and an ordered logistic model to impute breakfast consumption at age 17. Covariates included in the imputation models are the same included in the FE regression except for physical activity. FE confidence intervals are calculated using cluster standard error at the individual level. FE estimates are calculated using survey weight to account for attrition at age 17. We report 95% confidence intervals in brackets. ***, ** and * denote statistical significance at 1%, 5% and 10% levels respectively. ^a^ Indicates the lag value of the variable.
